# Astrocyte-derived CXCL10 exacerbates endothelial cells pyroptosis and blood–brain barrier disruption via CXCR3/cGAS/AIM2 pathway after intracerebral hemorrhage

**DOI:** 10.1038/s41420-025-02658-8

**Published:** 2025-08-08

**Authors:** Wenqianjun Sheng, Zhangyi Wu, Jingyan Wei, Jun Wang, Shengfan Zhang, Zhiquan Ding, Jinhao Zhong, Dexian Deng, Zhenzhong Zhong, Yunong Yin, Yulong Li, Qinghua Wang

**Affiliations:** 1 Neurosurgery Center, Neurotrauma Intensive Care Unit, Zhujiang Hospital, Southern Medical University, Guangzhou, China; 2 The Second School of Clinical Medicine, Zhujiang Hospital, Southern Medical University, Guangzhou, China; 3 Foshan Hospital of Traditional Chinese Medicine, the Eighth Clinical Medical College of Guangzhou University of Chinese Medicine, Foshan, Guangdong China

**Keywords:** Blood-brain barrier, Cell death in the nervous system

## Abstract

Intracerebral hemorrhage (ICH) is a devastating disease that disrupts the blood–brain barrier (BBB), triggers inflammation, and leads to subsequent neurological deficits. Although the CXC chemokine receptor 3 (CXCR3) and its ligand CXCL10 are implicated in regulating inflammation, the specific role and mechanism of CXCR3 in ICH-induced BBB disruption remain unclear; furthermore, the involvement of the cGAS/AIM2 signaling pathway in endothelial pyroptosis after ICH needs further investigation. This study elucidates that activation of the CXCR3/CXCL10 axis exacerbates disruption of BBB integrity via the cGAS/AIM2 pathway following ICH. Utilizing a type IV collagenase-induced ICH model, we evaluated the therapeutic efficacy of the CXCR3 inhibitor AMG487. Results demonstrated that ICH induced the upregulation of CXCR3 and CXCL10, peaking at 24 h; immunofluorescence co-localization indicated CXCR3 was primarily localized to endothelial cells, while CXCL10 originated mainly from endothelial cells and astrocytes. AMG487 treatment improved neurological deficits and attenuated BBB disruption after ICH. Furthermore, exogenous CXCL10 activating CXCR3 upregulated the expression of cGAS/STING and pyroptosis-related proteins in vivo and vitro ICH models. However, inhibiting CXCR3 reversed the poor effects induced by CXCL10. Inhibition of the cGAS/AIM2 signaling pathway using A151 effectively reduced vascular endothelial pyroptosis and BBB disruption. In a co-culture model of endothelial cells and astrocytes, depleting CXCL10 downregulated the expression of cGAS, STING, AIM2, and pyroptosis-related proteins and alleviated endothelial pyroptosis. This study demonstrates that inhibition CXCR3 preserves BBB integrity and improves neurological deficits after ICH by suppressing endothelial pyroptosis via the cGAS/AIM2 signaling pathway. These findings provide novel insights into ICH pathogenesis, proposing CXCR3 as a potential target for BBB disruption and AMG487 as a promising therapeutic strategy for ICH patients.

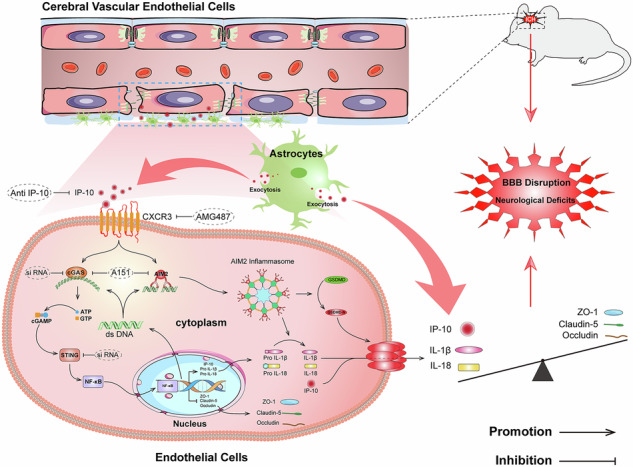

## Introduction

Intracerebral hemorrhage (ICH) represents 10–15% of all stroke cases and carries a mortality rate of 30–40% [1]. Survivors after ICH are often at high risk for disability and cognitive decline due to the lack of effective treatments. The compression injury caused by the hematoma results in primary brain damage. Secondary brain injury (SBI) is a complex of adverse reactions induced by the degradation products of hemoglobin. Activated microglial cells can disrupt the BBB, induce vasogenic edema, and lead to apoptosis of neurons [2]. High permeability of the BBB is one of the characteristic features of SBI [3]. Damage to the tight junction between endothelial cells leads to compromised integrity of the BBB, resulting in increased vascular permeability, cerebral edema, and impairment of neurological function [4]. High tight junction proteins can improve SBI and significantly enhance neurological function scores after ICH [5]. Therefore, preserving the integrity of the BBB is a promising strategy for treating ICH patients.

Chemokines and their receptor family are important members in activating immune cells and inducing inflammation [6]. CXCR3 belongs to the CXC chemokine receptor family and is primarily activated by CXCL9, CXCL10 and CXCL11 chemokines [7]. The activation of CXCR3 is associated with various neuroinflammatory diseases, and its role differs across different disease contexts [8]. Additionally, CXCR3 can regulate the efficiency of monocytes crossing BBB [9]. CXCL10 is one of endogenous ligand of CXCR3 and serves as a key pro-inflammatory signal [10]. Neuronal CXCL10/CXCR3 activation accelerates the efficiency of synaptic transmission, leading to brain hyperexcitation [11]. Clinical evidence shows that elevated CXCL10 is closely associated with a worse outcomes in ICH patients [12]. Moreover, CXCL10/CXCR3 decreased tumor angiogenesis and increased cell apoptosis in vivo and in vitro [13]. Although CXCL10 is closely related to ICH disease, its role in the BBB integrity damage after ICH is unclear.

In recent years, cytoplasmic DNA-sensing pattern have attracted much attention in nervous system diseases [14, 15]. cGAS recognizes exogenous and endogenous dsDNA in the cytoplasm, regardless of DNA sequence [16]. Upon binding to dsDNA, cGAS is a catalyst for the synthesis of cyclic GMP (cGAMP), which subsequently combines with and activates the STING. This activation promotes the nuclear translocation of IRF3, facilitating the transcription of inflammatory cytokines [17–19]. Activation of cGAS in microglia exacerbates the neuroinflammatory response in MPTP Parkinson mice [20]. Similarly, in Alzheimer’s disease, activation of cGAS exacerbates neuronal damage [15]. In addition, activation of the cGAS pathway also promotes neuron apoptosis via LINE-1 [21]. However, a large number of research focuses on the inflammatory process induced by microglia, but few research focuses on the role of cGAS in endothelial cells. Therefore, the cGAS activation in brain vascular endothelial cells after ICH requires exploration.

Caspase-1-dependent inflammasome signaling is increasingly recognized as a critical pathway in the pathogenesis of ICH [22]. AIM2 activation triggers the assembly of inflammasomes associated with various inflammatory responses elicited by sterile self-DNA. Specifically, AIM2 can assemble with Caspase-1 and ASC to form the AIM2 inflammasome, thereby exerting its biological effects, which promotes the release of IL-1β and IL-18 and induces pyroptosis in cells [23]. Besides directly triggering the assembly of the AIM2 inflammasome, dsDNA also participates in coordinating immune responses through type I interferons induced cGAS activation, indicating its involvement in multiple synergistic pathways [24–26]. Previous studies have shown that CXCL10-mediated CXCR3-positive macrophages may promote inflammatory responses in acute kidney injury through the cGAS/AIM2 pathway [27]. However, the role and mechanisms of CXCL10/CXCR3 and cGAS/AIM2 after ICH remain unclear. Therefore, we hypothesize that CXCL10 activates CXCR3 upregulating cGAS-STING and AIM2 signal, leading to endothelial cell pyroptosis and BBB disruption.

## Results

### The ICH model and mortality rate

The mortality rate for the mice was 5.64% (*n* = 15/266). None of the sham mice died and no significant difference in mortality rates between the treatment groups. Twenty-five mice were excluded from the study contributed to the absence of a hematoma in those with ICH.

### The time expression of endogenous CXCR3 increased following ICH

**Fig. 1:**
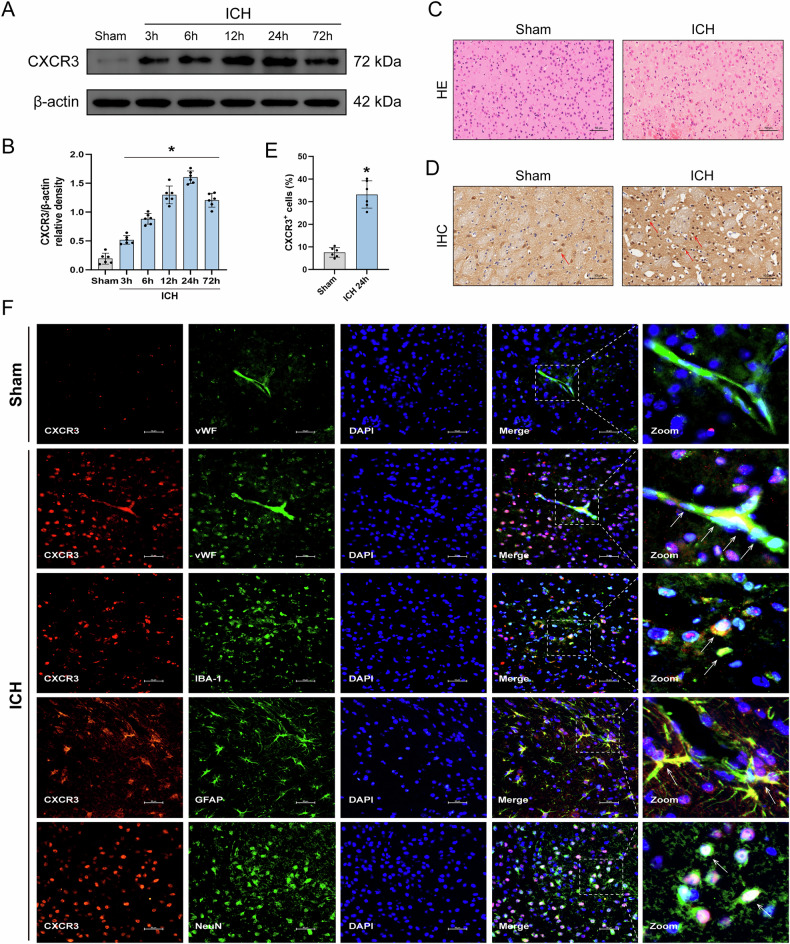
The expression and localization of CXCR3, and the tissue injury around the perihematomal area at different time points following ICH. **A** Representative western blot bands of CXCR3. **B** Quantitative analysis of CXCR3 protein expression, *n* = 6. **D**, **E** Immunohistochemical staining images and quantitative analysis of CXCR3 around the perihematomal tissue, *n* = 6. Scale bar= 50 μm. **C** Representative HE staining images of the perihematomal tissue, *n* = 6. Scale bar= 50 μm. **F** Representative immunofluorescence images of CXCR3 (red), vWF (green), NeuN (green), IBA-1 (green), GFAP (green), Nucleus (blue) around the perihematomal tissue, *n* = 6. Scale bar= 50 μm. *P < 0.05 vs. sham group.

We assessed CXCR3 in the tissue surrounding the hematoma through WB at 3, 6, 12, 24 and 72 h following ICH. CXCR3 protein levels were increased at 3 h and decreased at 72 h (Fig. 1A). Immunohistochemistry revealed CXCR3 increased in the ICH mice at 24 h (Fig. 1D, E). HE staining showed perihematomal tissue became loose with cell swelling and vacuoles after ICH (Fig. 1C). Immunofluorescence was used to examine the cellular localization of CXCR3. IF findings indicated that CXCR3 (red) was rarely expressed in the sham group and was observed within brain vascular endothelial cells (vWF), microglia cells (IBA-1), astrocytes (GFAP), and neurons (NeuN) that were stained green at 24 h post ICH (Fig. 1F).

### Inhibition of CXCR3 improved neurological dysfunction and decreased BWC post-ICH

**Fig. 2:**
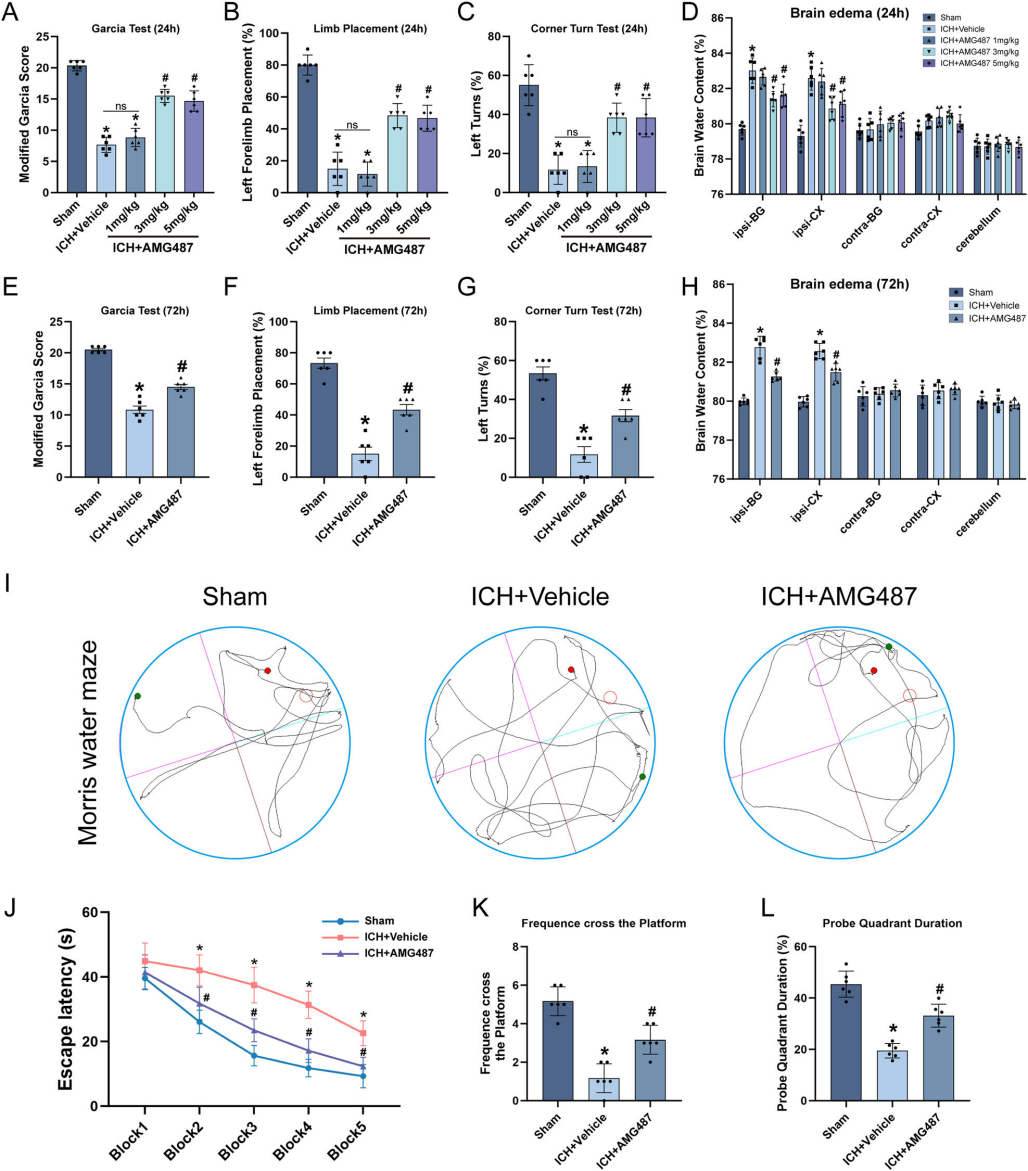
AMG487 improved neurological dysfunction and decreased brain edema following ICH. **A**–**C** The modified Garcia test, the left turn test and the forelimb placement test with different concentrations of AMG487 treatment at 24 h and 72 h post-ICH. **D** Brain water content after treatment with different concentrations of AMG487. **E**–**G** The modified Garcia test, the left turn test and the forelimb placement test with AMG487 treatment at 72 h post ICH. **H** Brain water content after treatment with AMG487 at 72 h post ICH. **I** Typical tracks of water maze exploration. **J**–**L** Escape latency, prob quadrant duration and frequence cross the platform, *n* = 6. *P < 0.05 vs. sham; ^#^*P* < 0.05 vs. ICH+vehicle; ns: no significant.

AMG487 (1, 3, 5 mg/kg) was administered at 30 min following ICH. Neurobehavioral tests were conducted at 24 and 72 h after ICH. Treatment with 3 mg/kg AMG487 was the most effective at improving neurological functions (Fig. 2A–C). BWC increased markedly in the injury brain at 24 h following ICH. However, ICH + AMG487 mice showed reduced brain water content (Fig. 2D).

**Fig. 3:**
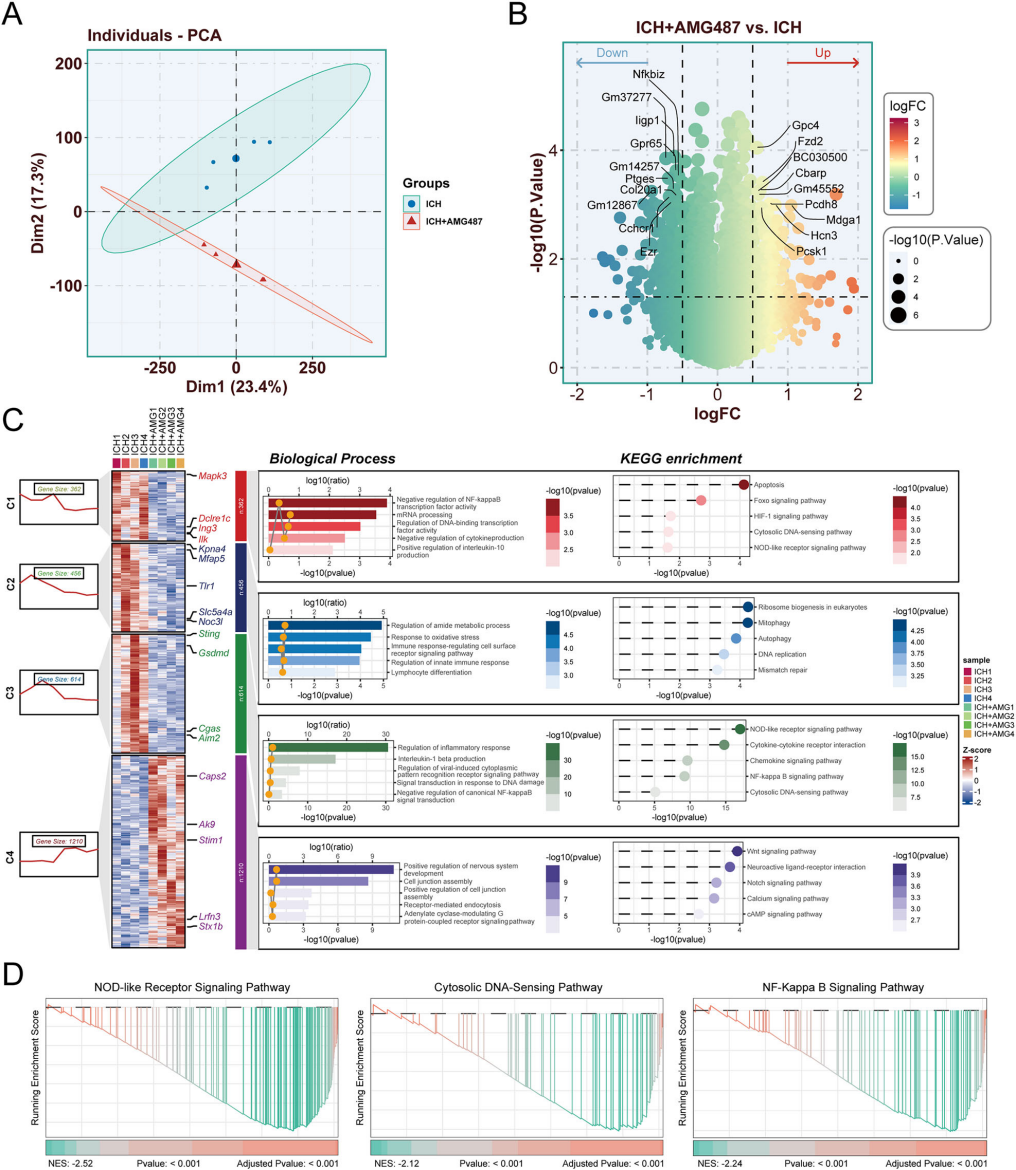
Differential expression gene and functional enrichment analysis of the ICH mice and ICH + AMG487 mice. **A** Principal components analysis of DEGs between ICH and ICH + AMG487 mice. **B** volcano plot of DEGs between the ICH and ICH + AMG487 mice. **C** The clustering results are presented in a trend heatmap. The left section displays the results of the trend clustering, while the middle section shows the heatmap of gene expression across the four clusters (C1, C2, C3, and C4). The right section is divided into three smaller parts from left to right: the first part indicates the number of genes contained in each cluster (C1, C2, C3, C4); the second part presents the biological processes associated with each cluster along with their corresponding log10 p-value; and the third part summarizes the KEGG enrichment analysis results for each cluster along with their corresponding log10 *p*-value. **D** GSEA enrichment analysis of NOD-like receptor signaling pathway, Cytosolic DNA-sensing pathway and NF-Kappa B signaling pathway.

To further evaluate the therapeutic efficacy of AMG487, neurological function was assessed 72 hours after ICH. AMG487 treatment improved the neurological deficits induced by ICH (Fig. 3E–G). BWC was increased in the injury brain at 72 h following ICH, but AMG487 treatment reduced BWC in perihematomal tissues (Fig. 2H).

A Morris Water Maze test examined the effects of AMG487 therapy on cognitive function following ICH. ICH mice had clear deficits in learning and memory, but AMG487 treatment enhanced their cognitive abilities (Fig. 2I–L).

### DEG identification and enrichment analysis after AMG487 treatment

To investigate CXCR3’s role in ICH pathogenesis, RNA-seq analysis was performed following intracerebroventricular AMG487 administration. Principal component analysis revealed distinct transcriptional profiles between ICH + AMG487 and ICH groups (Fig. 3A). We identified 446 differentially expressed genes (DEGs: 165 upregulated, 281 downregulated) (Fig. 3B). K-means clustering delineated four molecular subtypes with divergent expression patterns: C1-C3 showed downregulation (362, 456, and 614 genes, respectively) while C4 demonstrated upregulation (1210 genes) (Fig. 3C). Functional characterization revealed subtype-specific pathway associations: C1: Apoptosis-related processes; C2: Ribosome biogenesis; C3: NOD-like receptor/Cytosolic DNA-sensing pathways; C4: Wnt signaling. Previous research has indicated that the Wnt signaling pathway is closely related to the integrity of the vascular barrier function [28]. Notably, C3 exhibited the most significant expression changes, enriched with inflammasome components (cGAS, STING, AIM2, GSDMD) potentially influencing BBB dynamics. GSEA confirmed AMG487-mediated suppression of pro-inflammatory pathways (NOD-like receptors, DNA-sensing, NF-κB) (Fig. 3D). These findings suggest AMG487 modulates BBB integrity in ICH through cGAS-STING/AIM2 signaling regulation.

### AMG487 inhibition of CXCR3 reduce BBB disruption at 24 h post-ICH

**Fig. 4:**
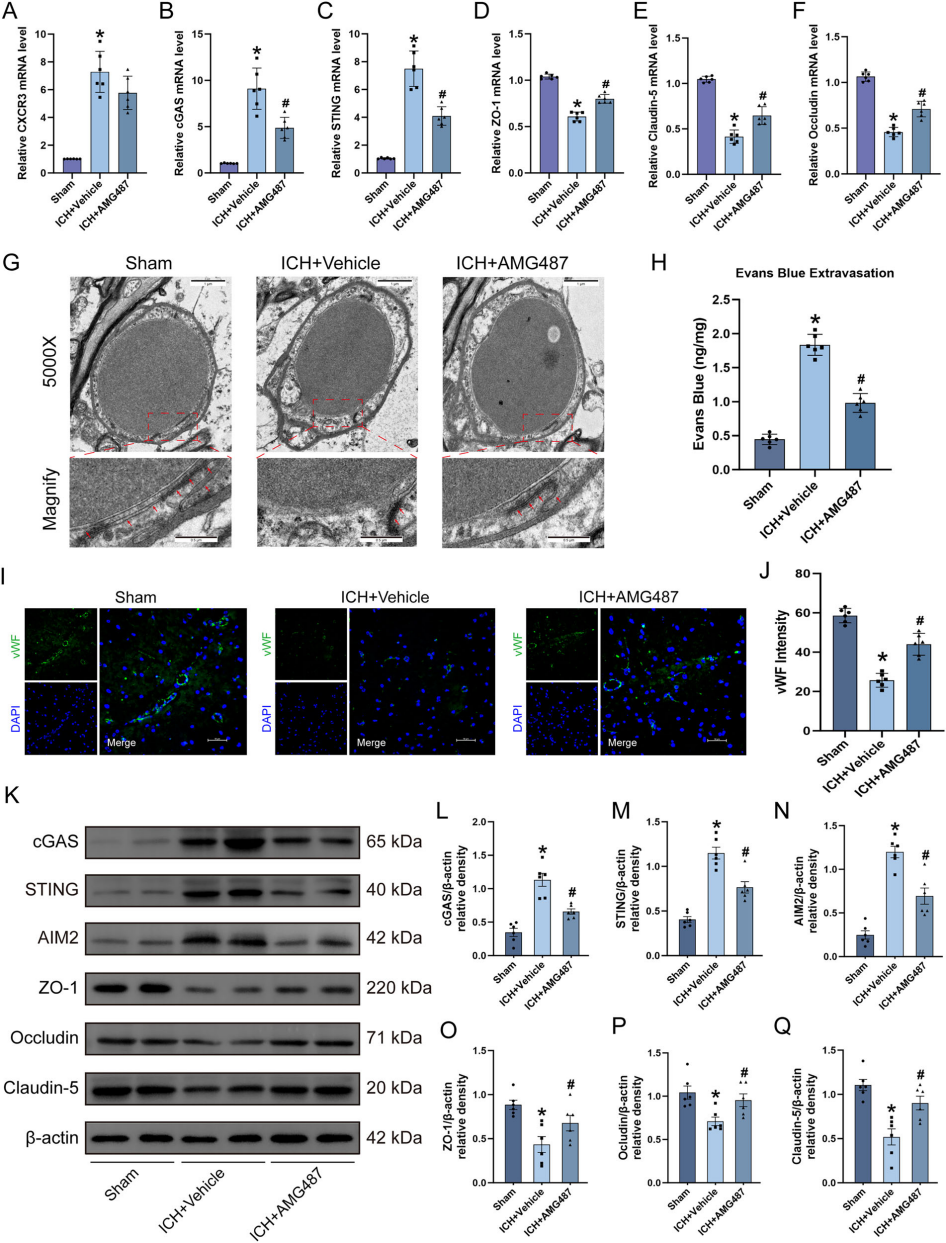
Intracerebroventricular administration of AMG487 preserves BBB integrity. **A**–**F** Quantification of CXCR3, cGAS, STING, ZO-1, claudin-5 and occludin relative mRNA level, *n* = 6. **G** Representative TEM images showing BBB disruption in perihematomal tissue at 24 h following ICH, *n* = 6. **H** Evans blue extravasation assay demonstrating dye leakage in perihematomal tissue at 24 h following ICH, *n* = 6. **I** Representative immunofluorescence images showing vascular endothelial cells (vWF, green) in perihematomal tissue at 24 h following ICH, *n* = 6. **J** Quantitative analysis of vWF fluorescence intensity. **K**–**Q** Representative western blot bands and quantitative analysis of cGAS, STING, AIM2, ZO-1, occludin, and claudin-5, *n* = 6. TEM scale bar =1 μm; Immunofluorescence scale bar =50 μm. *P < 0.05 vs. sham; ^#^*P* < 0.05 vs. ICH + vehicle.

To investigate CXCR3’s role in post-ICH neurological function, we evaluated BBB integrity through multimodal analyses. qRT-PCR revealed significant upregulation of CXCR3, cGAS, and STING mRNA alongside downregulation of tight junction components (ZO-1, occludin, claudin-5) in ICH (Fig. 4A–F). AMG487 treatment reversed these transcriptional alterations. TEM analysis demonstrated AMG487-mediated restoration of tight junction ultrastructural integrity following ICH-induced disorganization (Fig. 4G). Functional BBB assessment via Evans Blue extravasation showed AMG487 significantly attenuated ICH-induced permeability (Fig. 4H). Immunofluorescence revealed AMG487’s preservation of vascular endothelial integrity through increased vWF signal intensity in perihematomal tissue (Fig. 4I-J). Western blot analysis confirmed corresponding protein-level changes: ICH increased cGAS, STING, AIM2, and decreased tight junction proteins compared to sham group, while AMG487 treatment reversed these processes (Fig. 4K–Q). These findings collectively demonstrate CXCR3 inhibition ameliorates BBB disruption post-ICH through modulation of cGAS-STING signaling and tight junction preservation.

### Temporal expression and cellular localization of ligands for CXCR3 following ICH

**Fig. 5:**
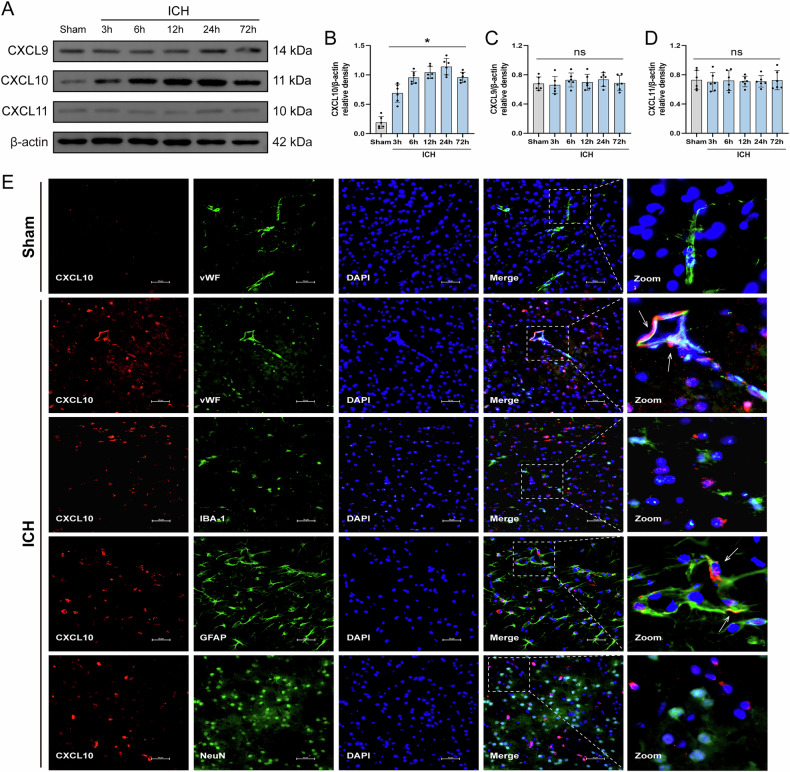
The expression and localization of CXCL10 around the perihematomal area at different time points following ICH. **A** Representative western blot bands of CXCL9, CXCL10 and CXCL11. **B**–**D** Quantitative analysis of CXCL9, CXCL10 and CXCL11 protein expression, *n* = 6. **E** Representative immunofluorescence images of CXCL10 (red), vWF (green), NeuN (green), IBA-1 (green), GFAP (green), Nucleus (blue) around the perihematomal tissue, *n* = 6. Scale bar= 50 μm. *P < 0.05 vs. sham; ns no significant.

To determine temporal expression profiles of CXCR3 ligands post-ICH, Western blot analysis revealed CXCL10 exhibited time-dependent upregulation (3–72 h) paralleling CXCR3 expression, while CXCL9/CXCL11 showed no significant temporal variation versus sham group (Fig. 5A–D). Immunofluorescence colocalization identified CXCL10 primarily localized to cerebrovascular endothelial cells (vWF) and astrocytes (GFAP), with minimal baseline expression in sham animals and marked induction post-ICH (Fig. 5E). These findings implicate CXCL10 as the principal CXCR3-activating ligand mediating blood–brain barrier disruption, predominantly derived from endothelial and astrocytic sources following ICH.

### Exogenous IP-10 aggravated mice neurological impairment, increased cerebral edema, and greater BBB disruption

**Fig. 6:**
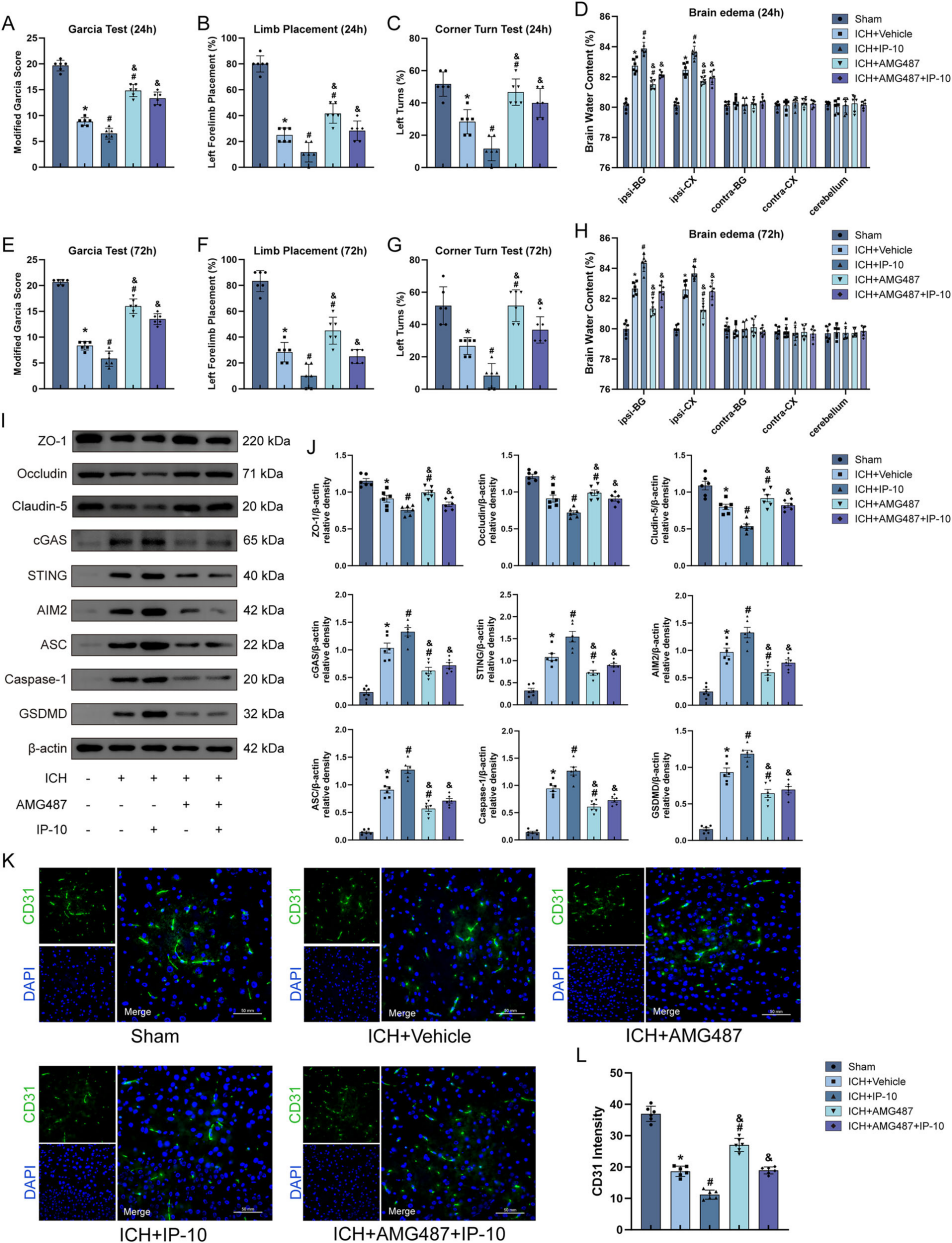
Exogenous IP-10 aggravates neurological deficits, exacerbates perihematomal brain edema, and decreases tight junction protein expression following ICH. **A**–**H** Neurological function assessment through the modified Garcia test, corner turning test, and forelimb placement test, along with brain water content measurement after ICH 24 h and 72 h, *n* = 6. **I**, **J** Representative western blot bands and quantitative analysis of ZO-1, occludin, claudin-5, cGAS, STING, AIM2, ASC, Caspase-1 and GSDMD. **K**, **L** Representative immunofluorescence images of CD31 and quantitative analysis of CD31 fluorescence intensity, *n* = 6. Scale bar = 50 μm. *P < 0.05 vs. sham; ^#^*P* < 0.05 vs. ICH + vehicle; ^&^*P* < 0.05 vs. ICH + IP-10.

To investigate IP-10/CXCR3 signaling in post-ICH pathogenesis, exogenous IP-10 was administered. IP-10 exacerbated neurological deficits and cerebral edema compared with ICH group at 24/72 h, effects reversed by CXCR3 antagonist AMG487 (Fig. 6A-H). Mechanistically, IP-10 amplified ICH-induced BBB disruption through coordinated downregulation of tight junction proteins (ZO-1/occludin/claudin-5) and upregulation of cGAS/STING signaling and pyroptosis relative proteins (Fig. 6I-J) and CD31 immunofluorescence (Fig. 6K-L). AMG487 pretreatment counteracted these IP-10-mediated pathological changes, demonstrating CXCR3/cGAS/STING axis-dependent exacerbation of BBB breakdown via pyroptotic mechanisms. These findings establish IP-10 as a critical driver of post-ICH neurovascular injury through CXCR3 activation.

### Inhibition CXCR3 attenuates endothelial pyroptosis via suppression of cGAS/STING pathway in vivo ICH models

**Fig. 7:**
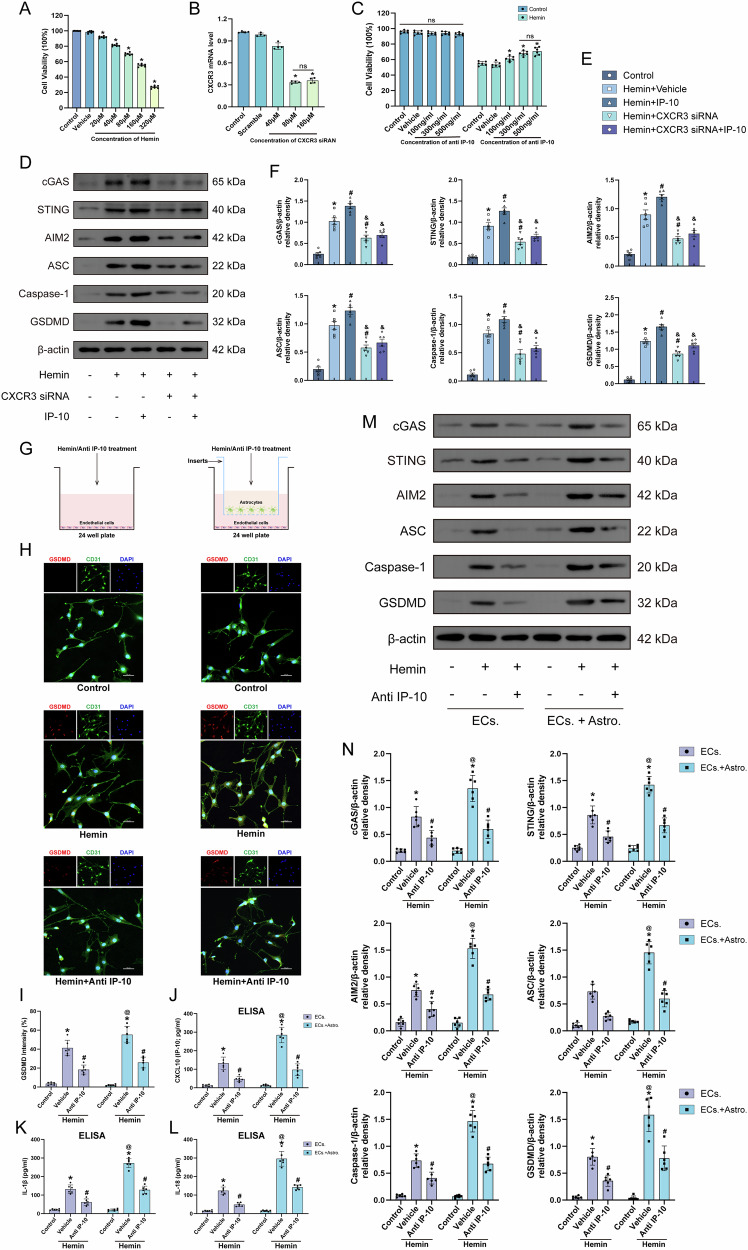
CXCR3 inhibition and neutralization CXCL10 attenuates endothelial cell pyroptosis induced hemin exposure. **A** CCK8 assay detection of endothelial cell viability stimulated by different concentrations of hemin. **B** Quantitative analysis of CXCR3 mRNA expression levels following treatment with varying concentrations of CXCR3 siRNA. **C** Optimal concentration of anti IP-10 for endothelial cell intervention by CCK8 assay, *n* = 6. **D**–**F** Representative western blot bands and quantitative analysis of cGAS, STING, AIM2, ASC, Caspase-1 and GSDMD, *n* = 6. *P < 0.05 vs. control; ^#^*P* < 0.05 vs. hemin+vehicle; ^&^*P* < 0.05 vs. hemin+IP-10. **G** Schematic diagram of endothelial cell-astrocyte co-culture system. **H**, **I** Representative immunofluorescence images of endothelial cells and quantification of GSDMD mean fluorescence intensity. **J**–**L** ELISA for CXCL10, IL-1β and IL-18 levels in medium, *n* = 6. **M**, **N** Representative western blot bands and quantitative analysis of cGAS, STING, AIM2, ASC, Caspase-1 and GSDMD, *n* = 6. *P < 0.05 vs. control; ^#^*P* < 0.05 vs. hemin+vehicle; ^@^*P* < 0.05 vs. ECs + hemin + vehicle; ns no significant.

To investigate whether inhibition of CXCR3 alleviated endothelial cell pyroptosis and improved BBB disruption through downregulation of the cGAS/STING signaling pathway. We cultured endothelial cells in vitro. To optimize experimental conditions in bEnd.3 cells, we conducted dose-response studies using varying concentrations of Hemin and CXCR3 siRNA. Cell viability was assessed using CCK8 method. Results showed that hemin exposed markedly reduced cell viability, with 160 μM identified as the optimal concentration (cell viability: 55.24 ± 2.23%; Fig. 7A). Furthermore, the effectiveness of CXCR3 knockdown was evaluated using qRT-PCR following 24-hour treatment with Hemin and different concentrations of CXCR3 siRNA (40, 80, 160 µM). Results confirmed that 80 µM CXCR3 siRNA achieved the most efficient transfection (Fig. 7B). CCK8 assays were conducted to identity the optimal concentration of anti IP-10, revealing that 300 ng/ml anti IP-10 was most effective in hemin-treated bEnd.3 cells (cell viability: 67.96 ± 4.73%; Fig. 7C). CXCR3 siRNA was used to transfect bEnd.3 cells or IP-10 was used to intervene the cells. WB results demonstrated that in comparison with the control group, a markedly elevated protein expression of cGAS, STING, AIM2, ASC, Caspase-1, and GSDMD in the hemin group (Fig. 7D–F). IP-10 treatment exhibited further increased in the levels of these proteins in comparison to the hemin group. However, CXCR3 siRNA markedly reduced expression of those proteins (Fig. 7D–F).

### CXCL10 secreted by astrocytes exacerbates endothelial cells pyroptosis

To delineate the cellular origin and functional impact of CXCL10 in ICH pathogenesis, in vitro endothelial monoculture and endothelial-astrocyte co-culture systems were established under hemin-simulated ICH conditions. Anti-IP-10 neutralizing antibody attenuated hemin-induced pyroptosis in both systems, as evidenced by reduced GSDMD fluorescence intensity (Fig. 7H–I) and suppressed IL-1β/IL-18 secretion via ELISA (Fig. 7J–L). Co-culture amplified hemin-triggered endothelial pyroptosis, correlating with enhanced cGAS/STING/AIM2 pathway activation and pyroptotic protein upregulation (Fig. 7M, N), effects reversed by CXCL10 inhibition. Mechanistically, astrocyte-derived CXCL10 in co-culture systems exacerbated endothelial pyroptosis by potentiating cGAS/STING signaling, confirmed via Calcein/PI staining (Fig. S2B, C). These results establish CXCL10 as a dual-origin mediator (endothelial/astrocytic) driving feedforward amplification of neurovascular injury through pyroptotic pathway activation.

### CGAS or STING knockdown attenuates poly(dA: dT)-induced endothelial cells pyroptosis

**Fig. 8:**
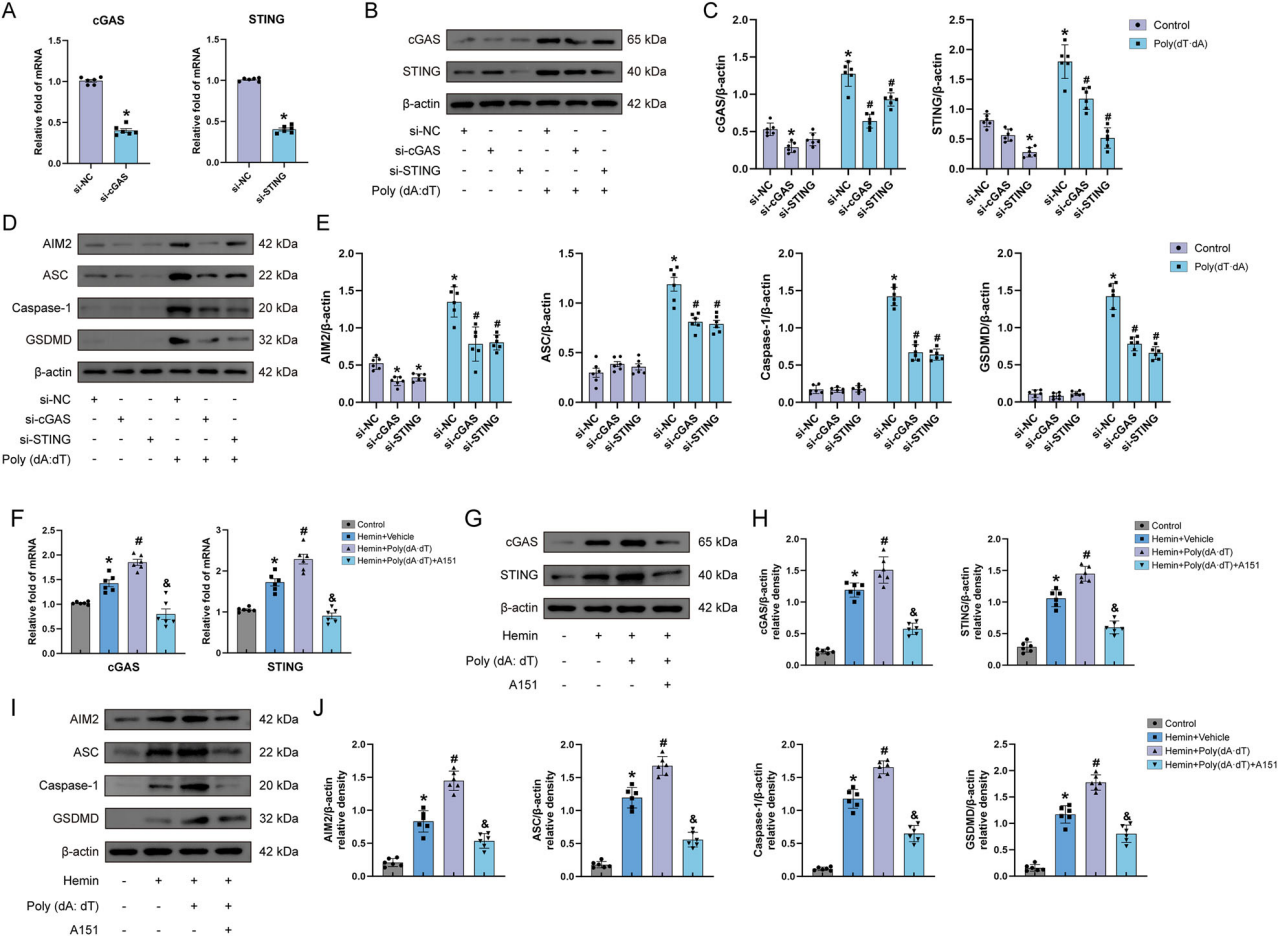
Inhibition of cGAS and STING respectively reduced endothelial cells pyroptosis. **A** Relative mRNA expression levels of cGAS and STING following transfection with cGAS or STING siRNA. **B**, **C** Representative western blot bands and quantitative analysis of cGAS and STING in bEnd.3 cells transfected with poly(dA:dT) following cGAS or STING knockdown, *n* = 6. **D**, **E** Western blot bands and quantitative analysis of AIM2 and pyroptosis-related proteins in bEnd.3 cells transfected with poly(dA:dT) after cGAS or STING knockdown, *n* = 6. *P < 0.05 vs. si-NC; ^#^*P* < 0.05 vs. si-NC+ Poly(dA:dT). **F** Relative mRNA expression levels of cGAS and STING following treatment with Poly(dA:dT) and A151, *n* = 6. **G**–**J** Representative western blot bands and quantitative analysis of cGAS, STING, AIM2, ASC, Caspase-1, GSDMD, *n* = 6. *P < 0.05 vs. control; ^#^*P* < 0.05 vs. hemin+vehicle; ^&^*P* < 0.05 vs. hemin + A151.

To elucidate endothelial-specific cGAS/STING contributions to BBB disruption post-ICH, we conducted siRNA-mediated knockdown of cGAS/STING in bEnd.3 cells, validated by qRT-PCR and western blot. Transfection with dsDNA analog poly(dA:dT) induced upregulation of cGAS/STING, AIM2, and pyroptotic markers, which was attenuated by cGAS/STING silencing (Fig. 8A–E). Notably, cGAS/STING depletion suppressed both dsDNA-induced AIM2 activation and pyroptotic protein expression, revealing pathway cross-talk. These findings demonstrate endothelial cGAS/STING signaling amplifies DNA-sensing cascades that drive pyroptotic BBB deterioration during ICH pathogenesis.

### A151 diminishes cGAS activation mediated by poly(dA:dT) and reduces AIM2-mediated pyroptosis in vitro

To delineate cGAS-STING/AIM2 contributions to BBB disruption, we tested the dual antagonist A151 in poly(dA:dT)-transfected, hemin-exposed bEnd.3 cells. A151 suppressed poly(dA:dT)-induced transcriptional upregulation of cGAS and STING (Fig. 8F). Western blot revealed hemin synergized with poly(dA:dT) to amplify cGAS, STING, AIM2, and pyroptotic proteins, while A151 attenuated these protein elevations (Fig. 8G–J). These results demonstrate cGAS-STING and AIM2 cooperatively drive DNA-sensing-mediated pyroptosis in endothelial cells, with A151’s dual inhibition highlighting pathway cross-talk in ICH-related BBB deterioration.

### A151 attenuates BBB disruption following ICH by inhibiting endothelial cell cGAS signaling and AIM2 Inflammasome-mediated pyroptosis

**Fig. 9:**
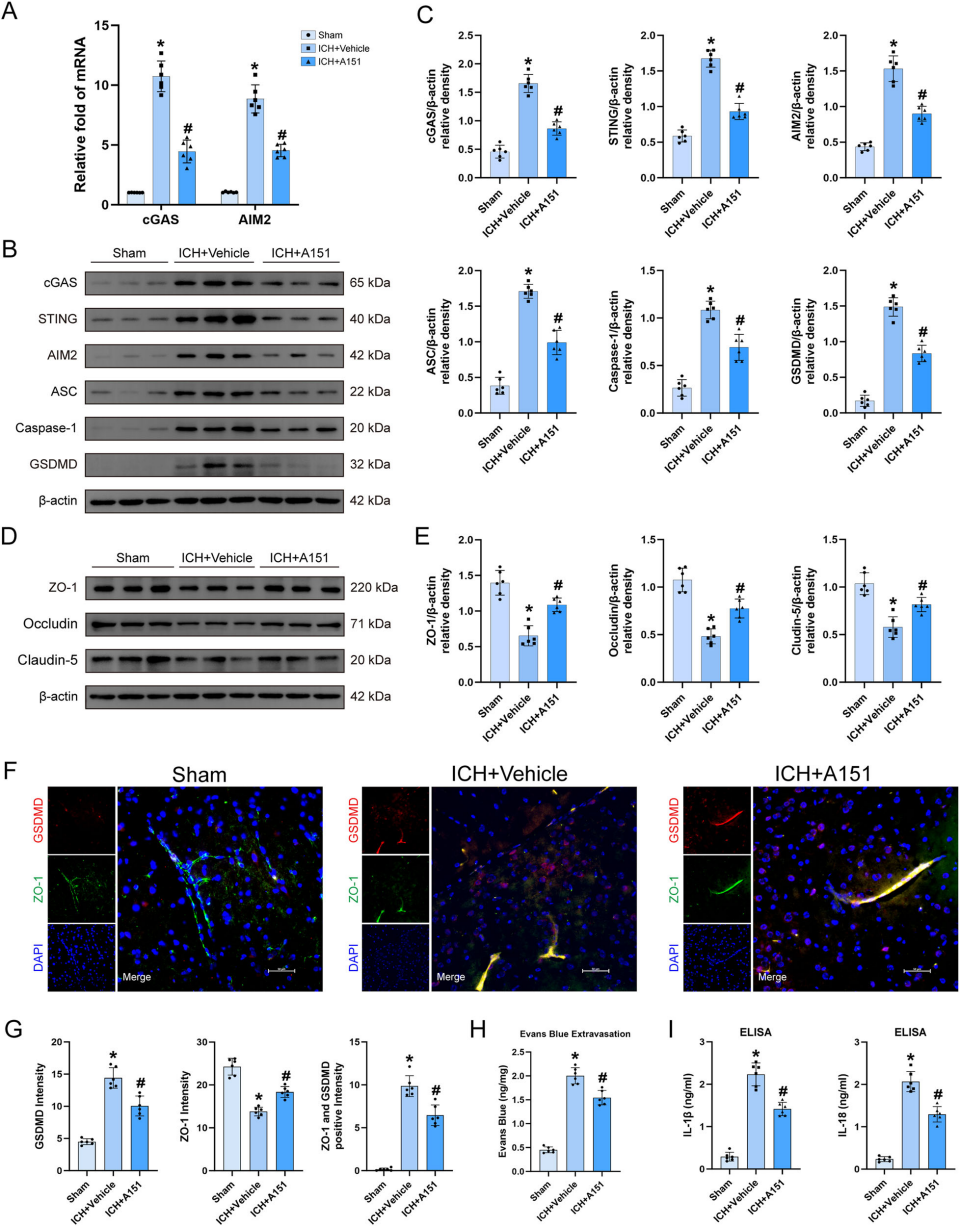
A151 treatment improved vascular endothelial cell integrity and mitigated BBB disruption in mice. **A** mRNA expression levels of cGAS and AIM2 in the perilesional tissue of the hemorrhagic brain, *n* = 6. **B**, **C** Representative western blot bands and quantitative analyses for cGAS, STING, AIM2, ASC, Caspase-1, and GSDMD in the perihematomal tissue, *n* = 6. **D**, **E** Representative western blot bands and quantitative analyses for tight junction proteins ZO-1, occludin, and claudin-5 in the perihematomal, *n* = 6. **F**, **G** Representative images of double immunofluorescence staining for ZO-1 and GSDMD in the perihematomal tissue 24 h after ICH, *n* = 6. Scale bar=50 μm. **H** Evans blue extravasation assay conducted at 24 h following ICH, *n* = 6. **I** ELISA for IL-1β and IL-18 levels in the perihematomal tissue, *n* = 6. *P < 0.05 vs. sham; ^#^*P* < 0.05 vs. ICH + vehicle.

To clarify AIM2’s role in ICH-induced BBB disruption, we assessed the dual antagonist A151 in vivo. A151 treatment in ICH mice suppressed cGAS/STING transcription and downregulated pyroptotic proteins, while restoring tight junction proteins (Fig. 9A–E). Immunofluorescence confirmed A151-mediated preservation of ZO-1 integrity and GSDMD suppression (Fig. 9F, G). Functional assays revealed reduced Evans Blue extravasation (Fig. 9H) and attenuated IL-1β/IL-18 levels (Fig. 9I) in A151-treated ICH mice. These findings demonstrate A151 mitigates BBB disruption by inhibiting AIM2 inflammasome-driven endothelial pyroptosis and cGAS/STING activation, highlighting its therapeutic potential in ICH-associated neurovascular injury.

## Discussion

Intracerebral hemorrhage is a severe type of stroke with few effective treatments. The BBB is a natural barrier between the CNS and peripheral circulation, serves as a critical safeguard for maintaining intracranial stability and preventing the entry of potentially neurotoxic substances [29]. Endothelial cells are the primary components of the BBB, and tight junctions, formed by ZO-1, claudin and occludin, are critical for maintaining the barrier’s integrity and function. The complex network of tight junctions between endothelial cells is essential for regulating the selective passage of molecules between the blood and the brain. Degradation of tight junction proteins has a crucial role on the integrity of the BBB, leading to compromised barrier function and increased permeability [30]. Thus, mitigating endothelial cell injury and tight junction protein degradation to the greatest extent possible represents a promising therapeutic strategy for minimizing neurological deficits following ICH.

This study aimed to elucidate the function of CXCL10/CXCR3 signaling in BBB following ICH. Furthermore, we explored how cGAS/STING signaling and AIM2 inflammasome mediate endothelial pyroptosis through cytosolic dsDNA recognition. Selective CXCR3 inhibitor AMG487 demonstrated protective effects against BBB permeability after ICH. Additionally, exogenous CXCL10-induced CXCR3 activation combined with AMG487 treatment resulted in attenuated BBB disruption and decreased expression of cGAS, STING, AIM2, ASC, Caspase-1, and GSDMD proteins.

Our findings revealed that: (1) CXCL10 and CXCR3 protein levels were significantly elevated in perihematomal tissue post-ICH, peaking at 24 h and declining after 72 h. CXCR3 mainly expressed in cerebral endothelial cells and neurons; CXCL10 was secreted by endothelial cells and astrocytes. (2) CXCR3 inhibition by AMG487 improved neurological outcomes, reduced cerebral edema, attenuated BBB disruption (validated by Evans Blue extravasation, TEM and IF experiments), and downregulated the expression of cGAS, STING, AIM2, ZO-1, occludin, and claudin-5. (3) RNA-sequencing analysis of perihematomal tissue at 24 h post-ICH with AMG487 treatment identified 446 differentially expressed genes. GO and KEGG revealed these genes were primarily involved in inflammatory responses and cytosolic DNA-sensing. GSEA further demonstrated significant downregulation of NOD-like signaling and cytosolic DNA-sensing pathways. (4) In vitro studies demonstrated that exogenous IP-10-induced CXCR3 activation combined with AMG487 treatment or CXCR3 siRNA transfection attenuated BBB disruption and decreased pyroptosis-related protein expression. (5) Neutralization of CXCL10 reduced endothelial cell pyroptosis. (6) Poly(dA: dT) transfection in bEnd.3 cells with cGAS and STING siRNA significantly reduced AIM2 inflammasome expression. (7) Simultaneous inhibition of cGAS and AIM2 using A151 resulted in decreased AIM2 inflammasome expression and attenuated BBB disruption. In conclusion, our findings demonstrate that CXCR3 activation contributes to BBB disruption, partially via cGAS/STING signaling and AIM2 pathways. Selective CXCR3 inhibition by AMG487 confers neuroprotection through suppressing endothelial pyroptosis, thereby preserving BBB integrity and improving neurological deficits following ICH.

Chemokines serve as critical regulators of target cell activity. They are categorized into four subfamilies: CXC, CC, CX3C, and XC [31]. Ligands of the CXCR3 receptor include CXCL9, CXCL10, and CXCL11, exhibit varied roles across different neurological disorders. CXCL10, but not CXCL9 or CXCL11, is significantly upregulated during the acute phase of ICH and is associated with poor prognosis, although its potential mechanisms remain unclear [8, 12]. In addition, CXCL10 is mainly secreted by endothelial cells and astrocytes [32]. Consistent with these findings, we found an increasing in CXCL10 expression in the perihematomal tissue following ICH in mice and co-located with endothelial cells and astrocytes. Furthermore, exogenous administration of CXCL10 exacerbated neurological deficits and the degree of cerebral edema in mice.

Previous research has indicated that CXCR3 regulated tumor migration, differentiation and exocytosis [33, 34]. In recent years, CXCR3 has also been the subject of growing interest in the context of non-neoplastic neurological disorders, where it exerts a pivotal influence on the modulation of inflammatory processes [35]. Our findings are consistent with prior research indicating that CXCR3 is predominantly expressed in vascular endothelial cells and neurons [36]. Growing evidence suggests that the interaction between CXCL10 and CXCR3 can induce apoptosis of vascular endothelial cells, leading to increased vascular permeability [37]. Based on these findings, we further explored functions of CXCL10/CXCR3 signaling in the disruption of the BBB after ICH.

AMG487, a selective CXCR3 inhibitor, has been demonstrated to effectively alleviate immune-induced inflammatory responses in vivo [38]. Research has shown that AMG487 can treat diabetic retinopathy in mice by inhibiting oxidative stress and endoplasmic reticulum stress [39]. In this study, we first used AMG487 as a protective agent to improve the integrity of BBB following ICH and explored its underlying mechanisms. We established a concentration gradient of AMG487 and screened the optimal dosage for treating ICH using behavioral function score tests, followed by the measurement of brain water content. The results revealed that AMG487 exhibited a significant protective effect on the BBB, which was validated by the reduction of EB extravasation and upregulation tight junction protein expression.

Subsequently, we further investigated the protective effect of AMG487-mediated CXCR3 inhibition on the integrity of the BBB. Through RNA-seq analysis, we found that 24 hours after treating ICH mice using AMG487, there was a significant downregulation of NOD-like receptor signaling and cytoplasmic DNA-sensing pathways in the perihematomal tissue. Extensive research has demonstrated that the NOD-like receptor family, a group of cytoplasmic pattern recognition receptors (PRRs), are crucial mediators of pyroptosis and play important roles in various neurological diseases [40]. Among these, AIM2 is a crucial member of the NOD-like receptor family and a vital participant in cellular pyroptosis. The ability of AIM2 to recognize dsDNA in conjunction with the cGAS-STING pathway and initiate inflammasome assembly underscores its importance in neuroinflammatory processes, highlighting its potential as a therapeutic target in various neurological disorders [41]. Previous studies have shown that cGAS/STING activation exacerbates microglia pyroptosis after ICH [42]. However, whether the cGAS/STING pathway and AIM2 inflammasome are involved in the pyroptosis of vascular endothelial cells following ICH remains unclear. In this study, we demonstrated that cGAS is effectively activated in response to specific damage-associated molecular pattern (DAMP) dsDNA released by pyroptotic endothelial cells after ICH, subsequently initiating downstream inflammatory responses through the STING signaling pathway. An in vitro model was developed to simulate dsDNA recognition in the context of ICH-induced injury, our findings demonstrated that cerebral vascular endothelial cells activate the cGAS-STING pathway, thereby inducing endothelial cell pyroptosis and exacerbating inflammatory responses. Inhibition of cGAS in cerebral vascular endothelial cells effectively reduced endothelial cell pyroptosis and preserved BBB integrity. These discoveries suggest that the cGAS-STING pathway activated in vascular endothelial cells contributes to the exacerbation of BBB disruption following ICH.

Notably, studies involving intraperitoneal administration of oligodeoxynucleotide (ODN) A151 have demonstrated its neuroprotective effects in models of ischemic stroke and Alzheimer’s disease due to its anti-inflammatory properties [38, 43]. Our experimental results indicate that A151 can effectively reduce pyroptosis of cerebral vascular endothelial cells under inflammation-induced conditions, thus protecting the BBB. Furthermore, A151 inhibits the activation of cGAS and STING, which attenuates vascular endothelial cell pyroptosis and improves the expression of tight junction proteins. These findings demonstrate that inhibition of the cGAS-STING pathway can mitigate BBB damage after ICH and improve neurological deficits.

In summary, our results showed that CXCR3 signaling plays a crucial impact in disrupting BBB integrity and that AMG487 can effectively mitigate this disruption following ICH. Our study demonstrates that CXCR3 activation exacerbates the pyroptotic response of vascular endothelial cells in the perihematomal tissue through the cGAS-STING and AIM2 pathways, ultimately leading to worsened neurological deficits in ICH mice. Therefore, targeting CXCR3 may represent a promising avenue for developing a therapeutic strategy to reduce BBB disruption.

However, our study has several limitations. Firstly, we have only investigated the role of CXCR3 in endothelial cells, and further studies are needed to explore its effects in neurons, microglia cells and astrocytes. Secondly, since estrogen modulates inflammatory pathways and affects BBB integrity, we used male mice exclusively to eliminate confounding effects from estrogen fluctuations [44]. The role of CXCR3 in female mice requires further investigation. Thirdly, given AMG487’s high molecular weight (603.59 Da) and limited BBB penetrability, we adopted intracerebroventricular administration following previous study [11]. Future studies need to address systemic delivery challenges to enable clinical translation of CXCR3-targeted therapies for ICH.

## Conclusion

Our study demonstrates that AMG487-mediated inhibition of CXCR3 maintains blood–brain barrier integrity and enhances both short-term and long-term neurological deficits after intracerebral hemorrhage by suppressing the cGAS-STING/AIM2 signaling pathway in mice. Therefore, AMG487 exhibits promising therapeutic potential and may be a viable therapeutic strategy for the treatment of ICH.

## Materials and methods

See the Supplementary Methods and Materials for details of the experimental design and methods.

## Supplementary information

Supplementary Methods and Materials

Supplementary Figure 1

Supplementary Figure 2

Supplementary Table 1

Supplementary Table 2

Uncropped original Western blot images

## Data Availability

All the data used in this study are available from the corresponding author upon reasonable request.

## References

[1] Sheth KN. Spontaneous intracerebral hemorrhage. N Engl J Med. 2022;387:1589–96. 36300975 10.1056/NEJMra2201449

[2] Qureshi AI, Mendelow AD, Hanley DF. Intracerebral haemorrhage. Lancet. 2009;373:1632–44. 19427958 10.1016/S0140-6736(09)60371-8 PMC3138486

[3] Wu X, Luo J, Liu H, Cui W, Guo K, Zhao L, et al. Recombinant adiponectin peptide ameliorates brain injury following intracerebral hemorrhage by suppressing astrocyte-derived inflammation via the inhibition of Drp1-mediated mitochondrial fission. Transl Stroke Res. 2020;11:924–39. 31902083 10.1007/s12975-019-00768-x

[4] Keep RF, Andjelkovic AV, Xiang J, Stamatovic SM, Antonetti DA, Hua Y, et al. Brain endothelial cell junctions after cerebral hemorrhage: changes, mechanisms and therapeutic targets. J Cereb Blood Flow Metab. 2018;38:1255–75. 29737222 10.1177/0271678X18774666 PMC6092767

[5] Burek M, Konig A, Lang M, Fiedler J, Oerter S, Roewer N, et al. Hypoxia-induced microRNA-212/132 alter blood-brain barrier integrity through inhibition of tight junction-associated proteins in human and mouse brain microvascular endothelial cells. Transl Stroke Res. 2019;10:672–83. 30617994 10.1007/s12975-018-0683-2 PMC6842347

[6] Vinader V, Afarinkia K. A beginner’s guide to chemokines. Future Med Chem. 2012;4:845–52. 22571610 10.4155/fmc.12.49

[7] Loetscher M, Gerber B, Loetscher P, Jones SA, Piali L, Clark-Lewis I, et al. Chemokine receptor specific for IP10 and mig: structure, function, and expression in activated T-lymphocytes. J Exp Med. 1996;184:963–9. 9064356 10.1084/jem.184.3.963 PMC2192763

[8] Zhou YQ, Liu DQ, Chen SP, Sun J, Zhou XR, Xing C, et al. The role of CXCR3 in neurological diseases. Curr Neuropharmacol. 2019;17:142–50. 29119926 10.2174/1570159X15666171109161140 PMC6343204

[9] Niu F, Liao K, Hu G, Moidunny S, Roy S, Buch S. HIV Tat-mediated induction of monocyte transmigration across the blood-brain barrier: role of chemokine receptor CXCR3. Front Cell Dev Biol. 2021;9:724970. 34527676 10.3389/fcell.2021.724970 PMC8435688

[10] Padovan E, Spagnoli GC, Ferrantini M, Heberer M. IFN-alpha2a induces IP-10/CXCL10 and MIG/CXCL9 production in monocyte-derived dendritic cells and enhances their capacity to attract and stimulate CD8+ effector T cells. J Leukoc Biol. 2002;71:669–76. 11927654

[11] Petrisko TJ, Bloemer J, Pinky PD, Srinivas S, Heslin RT, Du Y, et al. Neuronal CXCL10/CXCR3 axis mediates the induction of cerebral hyperexcitability by peripheral viral challenge. Front Neurosci. 2020;14:220. 32265633 10.3389/fnins.2020.00220 PMC7105801

[12] Landreneau MJ, Mullen MT, Messe SR, Cucchiara B, Sheth KN, McCullough LD, et al. CCL2 and CXCL10 are associated with poor outcome after intracerebral hemorrhage. Ann Clin Transl Neurol. 2018;5:962–70. 30128320 10.1002/acn3.595 PMC6093844

[13] Pan J, Burdick MD, Belperio JA, Xue YY, Gerard C, Sharma S, et al. CXCR3/CXCR3 ligand biological axis impairs RENCA tumor growth by a mechanism of immunoangiostasis. J Immunol. 2006;176:1456–64. 16424173 10.4049/jimmunol.176.3.1456

[14] Huang Y, Liu B, Sinha SC, Amin S, Gan L. Mechanism and therapeutic potential of targeting cGAS-STING signaling in neurological disorders. Mol Neurodegener. 2023;18:79. 37941028 10.1186/s13024-023-00672-x PMC10634099

[15] Govindarajulu M, Ramesh S, Beasley M, Lynn G, Wallace C, Labeau S, et al. Role of cGAS-sting signaling in Alzheimer’s disease. Int J Mol Sci. 2023;24:8151. 10.3390/ijms24098151 PMC10179704 37175853

[16] Sun L, Wu J, Du F, Chen X, Chen ZJ. Cyclic GMP-AMP synthase is a cytosolic DNA sensor that activates the type I interferon pathway. Science. 2013;339:786–91. 23258413 10.1126/science.1232458 PMC3863629

[17] Ablasser A, Goldeck M, Cavlar T, Deimling T, Witte G, Rohl I, et al. cGAS produces a 2’-5’-linked cyclic dinucleotide second messenger that activates STING. Nature. 2013;498:380–4. 23722158 10.1038/nature12306 PMC4143541

[18] Wu J, Sun L, Chen X, Du F, Shi H, Chen C, et al. Cyclic GMP-AMP is an endogenous second messenger in innate immune signaling by cytosolic DNA. Science. 2013;339:826–30. 23258412 10.1126/science.1229963 PMC3855410

[19] Hu X, Zhang H, Zhang Q, Yao X, Ni W, Zhou K. Emerging role of STING signalling in CNS injury: inflammation, autophagy, necroptosis, ferroptosis and pyroptosis. J Neuroinflammation. 2022;19:242. 36195926 10.1186/s12974-022-02602-y PMC9531511

[20] Ma C, Liu Y, Li S, Ma C, Huang J, Wen S, et al. Microglial cGAS drives neuroinflammation in the MPTP mouse models of Parkinson’s disease. CNS Neurosci Ther. 2023;29:2018–35. 36914567 10.1111/cns.14157 PMC10324349

[21] Gamdzyk M, Doycheva DM, Araujo C, Ocak U, Luo Y, Tang J, et al. cGAS/STING pathway activation contributes to delayed neurodegeneration in neonatal hypoxia-ischemia rat model: possible involvement of LINE-1. Mol Neurobiol. 2020;57:2600–19. 32253733 10.1007/s12035-020-01904-7 PMC7260114

[22] Li Y, Tu H, Zhang S, Ding Z, Wu G, Piao J, et al. P2Y6 receptor activation aggravates NLRP3-dependent microglial pyroptosis via downregulation of the PI3K/AKT pathway in a mouse model of intracerebral hemorrhage. Mol Neurobiol. 2024;61:4259–77. 38079109 10.1007/s12035-023-03834-6

[23] Hu B, Jin C, Li HB, Tong J, Ouyang X, Cetinbas NM, et al. The DNA-sensing AIM2 inflammasome controls radiation-induced cell death and tissue injury. Science. 2016;354:765–8. 27846608 10.1126/science.aaf7532 PMC5640175

[24] Lugrin J, Martinon F. The AIM2 inflammasome: Sensor of pathogens and cellular perturbations. Immunol Rev. 2018;281:99–114. 29247998 10.1111/imr.12618

[25] Chen D, Le SB, Hutchinson TE, Calinescu AA, Sebastian M, Jin D, et al. Tumor Treating Fields dually activate STING and AIM2 inflammasomes to induce adjuvant immunity in glioblastoma. J Clin Investig. 2022;132:e149258. 10.1172/JCI149258 PMC9012294 35199647

[26] Xu X, Fan H, Yang Y, Yao S, Yu W, Guo Z, et al. Virus-like particle-induced cGAS-STING activation and AIM2 inflammasome-mediated pyroptosis for robust cancer immunotherapy. Angew Chem Int Ed Engl. 2023;62:e202303010. 37040149 10.1002/anie.202303010

[27] Baatarjav C, Komada T, Karasawa T, Yamada N, Sampilvanjil A, Matsumura T, et al. dsDNA-induced AIM2 pyroptosis halts aberrant inflammation during rhabdomyolysis-induced acute kidney injury. Cell Death Differ. 2022;29:2487–502. 35739254 10.1038/s41418-022-01033-9 PMC9750976

[28] Song D, Ji YB, Huang XW, Ma YZ, Fang C, Qiu LH, et al. Lithium attenuates blood-brain barrier damage and brain edema following intracerebral hemorrhage via an endothelial Wnt/beta-catenin signaling-dependent mechanism in mice. CNS Neurosci Ther. 2022;28:862–72. 35343071 10.1111/cns.13832 PMC9062576

[29] Jia P, Peng Q, Fan X, Zhang Y, Xu H, Li J, et al. Immune-mediated disruption of the blood-brain barrier after intracerebral hemorrhage: Insights and potential therapeutic targets. CNS Neurosci Ther. 2024;30:e14853. 39034473 10.1111/cns.14853 PMC11260770

[30] Sun Q, Xu X, Wang T, Xu Z, Lu X, Li X, et al. Neurovascular units and neural-glia networks in intracerebral hemorrhage: from mechanisms to translation. Transl Stroke Res. 2021;12:447–60. 33629275 10.1007/s12975-021-00897-2

[31] Baggiolini M. Chemokines and leukocyte traffic. Nature. 1998;392:565–8. 9560152 10.1038/33340

[32] Cheng F, Wang C, Yan B, Yin Z, Liu Y, Zhang L, et al. CSF1R blockade slows progression of cerebral hemorrhage by reducing microglial proliferation and increasing infiltration of CD8 + CD122+ T cells into the brain. Int Immunopharmacol. 2024;133:112071. 38636374 10.1016/j.intimp.2024.112071

[33] Tokunaga R, Zhang W, Naseem M, Puccini A, Berger MD, Soni S, et al. CXCL9, CXCL10, CXCL11/CXCR3 axis for immune activation - a target for novel cancer therapy. Cancer Treat Rev. 2018;63:40–47. 29207310 10.1016/j.ctrv.2017.11.007 PMC5801162

[34] Chan TYH, Wong JSY, Kiang KM, Sun CWY, Leung GK. The duality of CXCR3 in glioblastoma: unveiling autocrine and paracrine mechanisms for novel therapeutic approaches. Cell Death Dis. 2023;14:835. 38104126 10.1038/s41419-023-06354-2 PMC10725418

[35] Wang F, Guo B, Jia Z, Jing Z, Wang Q, Li M, et al. The Role of CXCR3 in nervous system-related diseases. Mediators Inflamm. 2024;2024:8347647. 39429695 10.1155/2024/8347647 PMC11488998

[36] Van Raemdonck K, Van den Steen PE, Liekens S, Van Damme J, Struyf S. CXCR3 ligands in disease and therapy. Cytokine Growth Factor Rev. 2015;26:311–27. 25498524 10.1016/j.cytogfr.2014.11.009

[37] Qiao X, Zhang W, Zhao W. Role of CXCL10 in spinal cord injury. Int J Med Sci. 2022;19:2058–70. 36483597 10.7150/ijms.76694 PMC9724238

[38] Lu C, Ha T, Wang X, Liu L, Zhang X, Kimbrough EO, et al. The TLR9 ligand, CpG-ODN, induces protection against cerebral ischemia/reperfusion injury via activation of PI3K/Akt signaling. J Am Heart Assoc. 2014;3:e000629. 24721797 10.1161/JAHA.113.000629 PMC4187520

[39] Wang H, Li J, Zhong P, Wang S, Zhang L, Yang R, et al. Blocking CXCR3 with AMG487 ameliorates the blood-retinal barrier disruption in diabetic mice through anti-oxidative. Life Sci. 2019;228:198–207. 31039363 10.1016/j.lfs.2019.04.016

[40] Oladapo A, Jackson T, Menolascino J, Periyasamy P. Role of pyroptosis in the pathogenesis of various neurological diseases. Brain Behav Immun. 2024;117:428–46. 38336022 10.1016/j.bbi.2024.02.001 PMC10911058

[41] Lammert CR, Frost EL, Bellinger CE, Bolte AC, McKee CA, Hurt ME, et al. AIM2 inflammasome surveillance of DNA damage shapes neurodevelopment. Nature. 2020;580:647–52. 32350463 10.1038/s41586-020-2174-3 PMC7788527

[42] Yang G, Kantapan J, Mazhar M, Hu Q, Bai X, Zou Y, et al. Pretreated MSCs with IronQ transplantation attenuate microglia neuroinflammation via the cGAS-STING signaling pathway. J Inflamm Res. 2024;17:1643–58. 38504697 10.2147/JIR.S449579 PMC10949311

[43] Scholtzova H, Kascsak RJ, Bates KA, Boutajangout A, Kerr DJ, Meeker HC, et al. Induction of toll-like receptor 9 signaling as a method for ameliorating Alzheimer’s disease-related pathology. J Neurosci. 2009;29:1846–54. 19211891 10.1523/JNEUROSCI.5715-08.2009 PMC2699573

[44] Maggioli E, McArthur S, Mauro C, Kieswich J, Kusters DHM, Reutelingsperger CPM, et al. Estrogen protects the blood-brain barrier from inflammation-induced disruption and increased lymphocyte trafficking. Brain Behav Immun. 2016;51:212–22. 26321046 10.1016/j.bbi.2015.08.020

