# Supplementary material for: Astrocyte-derived CXCL10 exacerbates endothelial cells pyroptosis and blood–brain barrier disruption via CXCR3/cGAS/AIM2 pathway after intracerebral hemorrhage
Source: Cell Death Discov. 2025 Aug 8;11:373. doi: 10.1038/s41420-025-02658-8 (PMC12334743; doi:10.1038/s41420-025-02658-8)
Supplement: Supplementary file 1 — Supplementary Methods and Materials [file 41420_2025_2658_MOESM1_ESM.docx]

**Supplemental methods and materials**

**Experimental Design**

In order to investigate the impacts and mechanisms of CXCL10/CXCR3 in the pathogenesis of BBB damage after ICH, we meticulously designed five independent experiments. All animals were allocated into various experimental groups through a randomized selection process.

Experiment 1. To investigate the time-dependent changes of CXCR3, and cellular localization of CXCR3 in perihematomal tissue after ICH. Six groups were designed: sham, ICH (3h, 6h, 12h, 24h, 72h) groups, n=6. The expression of CXCR3 at those time points were detected using western blotting. Mice were allocation into 2 groups: sham and ICH groups, n=6. Immunofluorescence staining (IF) was conducted to investigate the colocalization of CXCR3. Immunohistochemical assessment of CXCR3 expression in the perihematomal tissue following ICH, n=6. Hematoxylin-eosin staining (HE) was used to assess the extent of damage in the perihematomal tissue, n=6 (Fig. S1A).

Experiment 2. To explore the effects of AMG487 on the BBB following ICH and to identify the optimal therapeutic dose, 5 groups was designed: sham, ICH + vehicle, ICH+ AMG487 (1.0mg/kg), ICH+ AMG487 (3.0 mg/kg), and ICH+ AMG487 (5.0 mg/kg) groups, n=6. Neurofunctional assessments and brain water content (BWC) measurements were performed 24 hours following ICH. The findings based on the neurological scores and BWC analysis indicated that a moderate dose of AMG487 (3.0 mg/kg) was the most effective in improving neurological functions and alleviating cerebral edema. Therefore, the moderate dose was selected as the treatment dose for follow experiments. To further observe the impact of AMG487 on brain water content and neurological outcomes at 72h following ICH, 3 groups were designed: sham, ICH+vehicle, and ICH+AMG487 groups, n=6. Following this assessment, the same group of mice received AMG487 (3 mg/kg) treatment for 24-28 days to conduct Morris Water maze, n=6 (Fig. S1B).

Experiment 3. Two groups were designed to conduct RNA-seq: ICH + vehicle group (n=4), and ICH + AMG487 (3.0 mg/kg) group (n=4). Tissue surrounding the hematoma was collected 24 hours post-ICH for RNA-seq. Differentially expressed genes (DEGs) were identified, and then enrichment with GO, KEGG, and GSEA databases to screen downstream pathways (Fig. S1B).

Experiment 4. To elucidate the potential neuroprotective effects of AMG487 on BBB integrity following ICH. Three groups were designed: sham, ICH+vehicle, and ICH+AMG487 groups, n=6. Western blot and qRT-PCR experiments were conducted, two experiment mice were both shared. The same group allocation for the Evans Blue (EB) extravasation assay, n=6. The same group allocation for the observation of BBB disruption using transmission electron microscope (TEM), n=6 (Fig. S1B).

Experiment 5. To clarify the trend of ligands of CXCR3 at different time points of ICH. The expression levels of CXCL9, CXCL10, CXCL11 were detected using western blot at 3h, 6h, 12h, 24h, 72h following ICH. Only CXCL10 was found to have a trend over time following ICH. IF was conducted to investigate the colocalization of CXCL10 in the perihematomal tissue following ICH, n=6 (Fig. S1A).

To investigate CXC10 activation CXCR3 in contributing to BBB disruption following ICH, five groups were designed: sham, ICH+vehicle, ICH+IP-10, ICH+AMG487, and ICH+AMG487+IP-10 groups, n=6. Neurological function and BWC were assessed at 24h and 72h following ICH. WB was conducted to analyze the proteins level of ZO-1, occludin, claudin-5, cGAS, STING, AIM2 and pyroptosis-relative proteins. Immunofluorescence was used to evaluate the expression of CD31 (Fig. S1C).

Experiment 6. To clarify whether CXCL10/CXCR3 disrupts the blood-brain barrier by aggravating endothelial cell pyroptosis and thereby disrupting the blood-brain barrier following ICH, we cultured bEnd.3 cerebrovascular endothelial cells in vitro. CCK-8 assays were conducted to determine the optimal concentration of hemin for stimulating bEnd.3 cells; concurrently, we evaluated cell viability after treatment with hemin and varying concentrations of CXCR3 siRNA. qRT-PCR experiment was conducted to screen the optimal transfection concentration of CXCR3 siRNA. CCK-8 assays were also employed to determine the optimal concentration of anti IP-10. The cells were categorized into five distinct groups: Control, hemin+vehicle, hemin+IP-10, hemin+CXCR3 siRNA, and hemin+CXCR3 siRNA+IP-10 groups, n=6. WB was performed to quantify the proteins level of cGAS, STING, AIM2, ASC, Caspase-1, and GSDMD. In addition, we cultured endothelial cells alone or used a co-culture system of endothelial cells and astrocytes stimulated using hemin and anti IP-10. Based on different treatments. The cells were categorized into three groups: Control, hemin+vehicle, hemin+anti IP-10 groups. WB, IF, PI and ELISA were conducted to assess endothelial cell pyroptosis level (Fig. S1D).

Experiment 7. To investigate the role of cGAS and AIM2 signaling in endothelial cells and BBB integrity, we used Poly(dA: dT) to simulate the dsDNA released into the cytoplasm following ICH. Using cGAS siRNA and STING siRNA transfected bEnd.3 cells, respectively. Initially, two major groups were designed: no Poly(dA: dT) and with Poly(dA:dT) groups. The primary group was subdivided into three: Control, cGAS siRNA, and STING siRNA, n=6. Within the second primary group was further divided into Poly(dA: dT) + si NC, Poly(dA:dT) + cGAS siRNA, and Poly(dA: dT) + STING siRNA groups. WB and qRT-PCR analysis were utilized to evaluate mRNA and protein expression levels of AIM2, ASC, Caspase-1, and GSDMD, respectively. The bEnd.3 cells were subjected to a treatment regimen comprising hemin and Poly(dA: dT), and the effects of cGAS and STING inhibition were investigated using the specific inhibitor A151. The experimental design consisted of four distinct groups: Control, hemin+vehicle, hemin + Poly(dA: dT), and hemin + Poly(dA: dT) + A151 groups, n=6. WB was performed to detect pyroptosis proteins (Fig. S1E).

In in vivo experiments, three groups were designed to investigate the role of A151 in ICH: sham, ICH + vehicle, and ICH + A151 groups, n=6. WB assessed pyroptosis protein expression and qRT-PCR was performed to quantify the cGAS and AIM2 mRNA level. Additionally, tight junction proteins were evaluated to examine the impact on BBB integrity. Immunofluorescence was used to explore the expression of ZO-1 and GSDMD. Moreover, enzyme-linked immunosorbent assay (ELISA) evaluated the expression of IL-1β and IL-18, key mediators of inflammation (Fig. S1F).

**Materials and methods**

**Animals**

All C57BL/6 mice, aged 10 weeks and weighing approximately 25 g to 30 g, were acquired from Sja Biotechnology (Guangdong, China). The animals were accommodated at specific pathogen-free environment where they had access to adequate food and water, and live with suitable temperature, humidity, and regular light-dark cycles. The procedures used in this experiment have been approved by the IACUC and are in line with guidelines set forth by Zhujiang Hospital of Southern Medical University (Animal Ethics No. LAEC-2024-301).

**ICH model**

As previous described ^1^, the induction of the experimental ICH model in this study was carried out with collagenase IV (Sigma, USA). Under anesthesia maintained by 1.0–1.5% isoflurane and supported with oxygen. Once mice anesthetized and secured, then the coordinates for the injection site were established by aligning the fontanelle and marking anterior1 mm and aside 2 mm of the fontanelle. A skull drill was then used to create a bone window with a diameter of 1.0 mm at the designated puncture site. Using of 0.04 U collagenase injected into the brain. The depth was about 3 mm. After injection, the bone was sealed with wax; the skin was stitched. The wound was disinfected with iodine and the mouse placed on a warming pad to monitor for recovery. The sham mice received an equal volume of saline.

**Drug Administration**

AMG487, a selective antagonist of the CXCR3; recombinant mouse CXCL10 (IP-10), imitation endogenous CXCL10 activity; and A151 (ODN TTAGGG), a selective antagonist of cGAS and STING were obtained from Med Chem Express (MCE, China). In vivo experiment, AMG487 was intracerebroventricular injection at three different doses of 1, 3, and 5 mg/kg, 30 minutes after the induction of ICH. Based on neurological functional scoring, 3 mg/kg AMG487 was determined the most effective concentration. In vivo experiment, the concentration of 1μg/2μL IP-10 administered intracranially ^2^; in vitro experiment, 100ng/mL IP-10 exposed to bEnd.3 cells ^3^; Based on the CCK8 results, 300ng/mL anti IP-10 for neutralization of CXCL10 in vitro experiment. 300 μg/0.2μl A151 was treated following ICH ^4^. In in vitro experiments, hemin was exposed to bEnd.3 cells at concentrations of 20, 40, 80, 160, and 320 μM, with the optimal concentration determined to be 160 μM. When transfecting bEnd.3 cells with CXCR3 siRNA at concentrations of 10, 20, and 40 μM, a concentration of 20 μM CXCR3 siRNA showed the best transfection efficiency. Poly(dA: dT) at 2 μg/ml, a DNA synthetic polymer capable of activation cGAS and AIM2; cGAS siRNA, and STING siRNA at 100 μM were used for transfection of bEnd.3 cells, while A151 was treatment to bEnd.3 cells at a concentration of 3 μM ^4^.

**Intracerebroventricular Injection**

As previous described^1^, anesthesia was induced using 1.0–1.5% isoflurane with oxygen supplementation. The head of each anesthetized mouse was securely positioned in a stereotaxic instrument head holder. The mice's head hair was then removed, and its skin disinfected using an iodine complex, followed by a longitudinal incision to fully expose the fontanelle. Taking the anterior fontanelle as a reference, a 1mm cranial hole was drilled 1mm laterally and 0.2mm posteriorly using a skull drill. The needle was inserted 2.25mm into the ventricle. AMG487 was administered via microinjection followed by a 5-minute pause to minimize backflow. The needle was then withdrawn. The procedure was finalized by sealing the cranial hole with bone wax.

For behavioral experiments that require daily drug infusion, a guide cannula for intracerebroventricular injection was implanted on the left side for repeated direct intraventricular injections. Under anesthesia maintained by 1.0–1.5% isoflurane, their heads were secured in a stereotaxic frame. As previously described ^5^, the skull was exposed and holes were drilled at the puncture site for cannula placement. Additionally, two holes were drilled on either side of the puncture site to provide anchor points for dental cement. The cannula was inserted into the lateral ventricle for drug administration, and after the infusion was complete, the cannula cap was slowly inserted and tightened in preparation for future injections.

**Cell culture and In vitro ICH model**

Mouse brain endothelial cell (bEnd.3, CL-0598) was sourced from Procell Life Science & Technology (Wuhan, China) and cultured according to the specifications outlined by the company. The culture medium consisted of DMEM supplemented with 100 U/ml penicillin, 100 μg/ml streptomycin (Solarbio, Beijing, China), and 10% fetal bovine serum with glucose. Cells were placed at 37°C in a 5% CO2 atmosphere, seeded at a density of approximately 5 × 10^5^ cells per well in a 6-well plate, and exposed to 160 μM hemin to simulate a hemorrhagic environment.

**Primary astrocyte extraction and culture**

As previous study described ^6^, cortical astrocytes were isolated from neonatal mice within 24 hours post-delivery. Following meninges removal, the dissected cerebral cortex underwent enzymatic dissociation using 2.5% trypsin (Gibco, USA) at 37°C for 15 min. The enzymatic reaction was quenched with DMEM growth medium supplemented with 10% fetal bovine serum (FBS; Gibco) and 1% penicillin-streptomycin cocktail. Cell suspensions were subsequently centrifuged (300g, 5 min) and plated in serum-enriched culture medium. Cells were maintained in a humidified atmosphere (5% CO2, 37°C) with medium replacement every 72 h. Astrocytic identity was confirmed through dual-immunofluorescence staining using specific markers: GFAP for astrocyte and IBA-1 for microglia (Fig. S2A).

**Western blot assay**

WB analysis was conducted compliance with the method previously reported ^7^. Perihematomal tissue was taken and placed in a centrifuge tube containing RIPA lysis buffer, to which protease and phosphatase inhibitors, as well as grinding beads, were added. The centrifuge tube was placed in a 4°C Lukas mill with parameters set to 70Hz/60s. After grinding, Centrifuge at 12,000 rpm for 15 minutes at 4°C. The upper layer was saved and boiled for 15 minutes with loading buffer. SDS-PAGE gel loaded with protein samples. After electrophoresed and then transferred to a PVDF membrane, which using 5% bovine serum albumin blocked for 2 hours, and exposed to primary antibody working solution at 4°C for 15-18 hours. The dilution ratio of the primary antibody and the manufacturer information are detailed in Table 1. The membrane was exposed to the appropriate secondary antibody for 1-2 hours. The membrane was probed with HRP substrate (Millipore, USA). Proteins were visualized with the Nine Alliance imaging system (UVItec, UK). Grey values were analyzed with Image J.

**Immunofluorescence Staining**

Three 8mm thick coronal brain sections were taken from each animal, and 10 μm thick slices were made at the basal ganglia with hematoma for staining. Twenty-four hours after successful induction of the intracerebral hemorrhage model, mice were anaesthetized and perfused with saline and 4% paraformaldehyde. Samples were fixed and dehydrated twice in 30% sucrose. Samples were made into 10 μm coronal slices with a cryostat (Leica, Germany). Primary antibodies were incubated at 4°C for 12 hours (Table 1 consistent of detailed primary antibodies). Brain slices were soaked in secondary antibodies for 2 hours. Nikon Ti2E microscope took pictures. Image J software was used to analyze images.

**Assessments of BBB permeability**

To assess BBB disruption, a 4-hour pre-euthanasia intraperitoneal injection of 0.25 ml of 2% Evans Blue dye (E2129; Sigma-Aldrich) was administered. After deep anesthesia, perfusion with pre-cooled PBS and brain tissue samples are homogenized in PBS (1ml/300g), followed by sonication. Subsequently, the homogenate is subjected to centrifugation 15,000 rpm at 4°C for 30 minutes, then incubate the supernatant with an equal volume of trichloroacetic acid (50%) overnight. On the following day, centrifugation was repeated at 4°C at 15,000 rpm for 30 minutes. the quantity of Evans Blue dye extravasation was quantified utilizing a spectrophotometer at 620 nm (Thermo Fisher, USA), employing a standard curve for calibration and normalization to the weight of the tissue.

**Neurobehavioral Tests**

the modified Garcia score consists of seven assessment items, which can be further divided into four items with scores ranging from 0 to 3 (spontaneous activity, symmetry of limb movement, body proprioception, and extending forelimbs) and three items with scores ranging from 1 to 3 (climbing, sensory response, and lateral turning). The total score, which reflects the overall neurological function, is the sum of these seven items, with a minimum of 3 and a maximum of 21 points, where a lower score indicates more severe neurological deficits. The score criteria for each item : (1) spontaneous activity was assessed by observing the mouse's self-initiated movements in a new cage for 5 min; (2) body proprioception was evaluated by observing the angle and range of lateral turning when the tail was lifted; (3) sensory response was determined by touching the whiskers on both sides with a cotton swab and observing the reaction; (4) extending forelimbs was assessed by observing the extension of the forelimbs when the tail was lifted; (5) axial sensation was evaluated by lightly touching both sides of the body with a stick and observing the reaction; (6) symmetry of limb movement was assessed by observing the movement of the four limbs when the tail was lifted; and (7) climbing ability was evaluated by observing the mouse's active climbing on an inclined surface.

A corner test was conducted by setting up two partitions at a 30-degree angle to the table surface. The mouse was gently encouraged to walk towards the corners formed by the two partitions. When the mouse entered the corner, it instinctively chose to turn it. Each mouse was tested 10 times, with a 30-second interval between trials. Given the potential for damage to the right basal ganglia in the experimental model, the focus was on recording the frequency of left turns made by the mouse during 10 trials, which was then expressed as a percentage.

The forelimb placement test assesses the motor response of the mouse forelimbs to tactile stimulation. After ensuring that the mouse's limbs were in a relaxed, non-tensed state, the mouse was slowly brought to the edge of a table and one of its whiskers lightly touched the table surface. The researcher then observed whether the mouse forelimb extended naturally towards the table edge when the whisker contacted the surface. Each mouse was tested 10 times, with a 30-second interval between trials, and the frequency of successful forelimb extension towards the table edge was recorded. Given the potential damage to the right basal ganglia in the experimental model, only the frequency of left forelimb extension was calculated and expressed as a percentage.

The Morris water maze experiment was conducted between days 24 and 28 following ICH. As previous study described ^6^, a 120 cm diameter pool was filled with white liquid, forming four quadrants. A 5 cm platform was positioned at the center of one quadrant. During the first 5 days of training, a submergence level of approximately 1 cm was recorded for the platform, and mice were placed into the pool from four different starting positions, with each trial lasting 60 seconds. On the sixth day, the platform was taken out, and the mice started from a fixed location. A camera recorded the mice's movements, and software determined the time needed to locate the platform during training, the amount of time spent within the specified quadrant after platform removal, and times the mice crossed the platform.

**Brain water content**

According to previous reported ^8^, BWC was evaluated at 24h and 72h. After deep anesthesia, mice brain tissue was immediately harvested following perfusion with PBS. The sample was then quickly separated into the ipsilateral and contralateral cortex, basal ganglia, and cerebellum. Weighing each compartment on an electronic balance and accounting for moisture. Each tissue was then wrapped in aluminum foil and dried for 24 hours at 100°C to determine the dry weight, which was then calculated with the formula: Brain Water Content (%) = (Wet Weight - Dry Weight) / Wet Weight × 100%.

**RNA-seq**

As previous described ^9^, total RNA was extracted from ICH and ICH+AMG487 brain tissues using Trizol reagent. The quality of the RNA was then checked using the Nano-100 system, and samples with a RIN value greater than 7.0 were chosen for the library construction. The mRNA was purified from 5 µg of total RNA using Dynabeads Oligo (dT) and fragmented with divalent cations at 94°C for 6 minutes. The mRNA was then converted to cDNA using the FastKing gDNA Dispelling RT SuperMix kit (TianGen Biotech). Second-stranded DNA synthesis was then facilitated using E. coli DNA polymerase I, RNase H, and dUTP Solution (Thermo Fisher). PCR was performed with a SYBR Green kit (TianGen) on a BIO-RAD C1000 Touch Thermal Cycler.Paired-end sequencing (2 x 150 bp) was conducted on an Illumina Novaseq™ 6000. The raw reads were trimmed for adapters and low-quality bases using Cutadapt (with a Phred quality cutoff of 20), and quality was reassessed with FastQC. Differentially expressed genes (DEGs) were identified using DESeq2, with DEGs defined as those with log2FoldChange > 0.5 and P.value < 0.05. Volcano plots were created with ggplot2 (R version 4.2.1), and DEGs were analysed using the complete-linkage method. Gene Ontology (GO), Kyoto Encyclopedia of Genes and Genomes (KEGG) and Gene Set Enrichment Analysis (GSEA) pathway analyses were conducted to annotate the potential functions of DEGs. The data were deposited in the CNGB Sequence Archive (CNSA) of the China National GeneBank DataBase (CNGBdb) under accession number SUB057791 (<https://db.cngb.org/cnsa/>).

**qRT-PCR**

As previously described ^1^, perihematomal tissue was collected 24h following ICH induction and the AG RNAex Pro RNA reagent was used to extract total RNA. A SYBR Green Pro Taq HS reverse transcription kit was used to generate cDNA. A Bio-Rad system was used for qRT-PCR analysis. GAPDH was served as an internal control. The 2^-ΔΔCt^ method was used to analyze the relative expression levels of the target genes. The primer sequences are shown in Table 2.

**Hematoxylin and eosin staining**

As previously described ^10^, after deep anesthesia, perfusion was performed using pre-cooled 0.1 M PBS (pH 7.4) until the liver turned white, at which point the perfusion solution was switched from PBS to 4% paraformaldehyde. The brain tissue was immersed in 4% paraformaldehyde for 24 h at 4°C. After dehydration and paraffin embedding, continuous coronal sections of 4 μm thickness were obtained. Briefly, brain sections were deparaffinized, rehydrated, and stained with hematoxylin and eosin. The staining duration was adjusted based on the desired staining intensity. Images were captured using an optical microscope (Leica, Germany), and the observers were blinded to the experimental groups.

**Immunohistochemical staining**

As previously described ^11^, after dewaxing, rehydration and antigen retrieval, the sections were incubated with 3% hydrogen peroxide for 15 minutes. Then, 5% goat serum was added before the primary antibody was added to the working solution and incubated at 4°C for 12–15 hours. The same antibodies were used for immunohistochemical and IF staining of CXCR3. Sections were then incubated with biotinylated goat anti-rabbit and horseradish peroxidase-streptavidin reagent for 15 minutes each. Finally, 3,3'-diaminobenzidine was used to stain the sections. The sections were observed and images were taken using a microscope (3D HISTECH, China) by personnel blinded to the group.

**Transmission electron microscopy**

After deep anesthesia, the mice perfused with PBS, followed by a secondary perfusion using a 0.1-M Sodium Cacodylate Buffer with 2% Paraformaldehyde (PFA) and 2% Glutaraldehyde. The tissue surrounding the hematoma was meticulously dissected and incubated in fixative solution for a period of 24 hours. Subsequently, the tissue was post-fixed with 1% osmium tetroxide and 1% potassium ferrocyanide, followed by en bloc staining using 2% uranyl acetate prior to resin embedding. Ultra-thin slices were prepared using an RMC MT-X ultramicrotome and subsequently treated with a 2% uranyl acetate and 1% lead citrate solution. The sections were then analyzed and imaged using transmission electron microscope (JEOL, Japan), operated at an accelerating voltage of 80 kV.

**ELISA analysis**

As previously described ^1^, ELISA kits for CXCL10, IL-1β and IL-18 were purchased from R&D Systems (Minneapolis, USA). Briefly, the mice were deeply anesthetized and perfused transcardially with PBS, and brain tissues were quickly harvested; The samples were homogenized using a Dounce homogenizer (Thermo Fisher Scientific, USA) in buffer containing a protease inhibitor cocktail (Nacalai Tesque, Japan). In vitro experiments, culture medium was collected for the detection of CXCL10, IL-1β and IL-18.

**Calcein/PI Staining**

As previously described ^12^, To determine the degree of endothelial cell pyroptosis, a calcein/PI cell viability assay kit was used to detect cell membrane permeability. Each sample was randomly photographed in 6 fields of view to count the average fluorescence intensity of cells (%) under a fluorescence microscope and quantified using ImageJ software.

**Statistical Analysis**

The statistical analyses were conducted using Prism 10.1.2 (GraphPad prism, USA). One-way ANOVA and Neuman-Keuls post hoc test were employed for the purpose of comparison. P＜0.05 was considered to be statistically significant. The number of samples (n) represents the times of replicates. The dimensions of the experimental samples are provided in the figure legends. All results are expressed as mean ± standard deviation. Furthermore, a two-way ANOVA was used for the analysis of the Morris water maze experiment data.

**References**

1 Li Y, Tu H, Zhang S, Ding Z, Wu G, Piao J *et al.* P2Y6 Receptor Activation Aggravates NLRP3-dependent Microglial Pyroptosis via Downregulation of the PI3K/AKT Pathway in a Mouse Model of Intracerebral Hemorrhage. Mol Neurobiol*.* 2023.

2 Hermans E C, Donega V, Heijnen C J, de Theije C G M & Nijboer C H. CXCL10 is a crucial chemoattractant for efficient intranasal delivery of mesenchymal stem cells to the neonatal hypoxic-ischemic brain. Stem Cell Res Ther*.* 2024; 15: 134.

3 Zhang Y, Gao Z, Wang D, Zhang T, Sun B, Mu L *et al.* Accumulation of natural killer cells in ischemic brain tissues and the chemotactic effect of IP-10. J Neuroinflammation*.* 2014; 11: 79.

4 Wu X, Yu N, Ye Z, Gu Y, Zhang C, Chen M *et al.* Inhibition of cGAS-STING pathway alleviates neuroinflammation-induced retinal ganglion cell death after ischemia/reperfusion injury. Cell Death Dis*.* 2023; 14: 615.

5 Terburg D, Scheggia D, Triana Del Rio R, Klumpers F, Ciobanu A C, Morgan B *et al.* The Basolateral Amygdala Is Essential for Rapid Escape: A Human and Rodent Study. Cell*.* 2018; 175: 723-735 e716.

6 Gao M, Song Y, Liu Y, Miao Y, Guo Y & Chai H. TNF-alpha/TNFR1 activated astrocytes exacerbate depression-like behavior in CUMS mice. Cell Death Discov*.* 2024; 10: 220.

7 Ding Z, Zhong Z, Wang J, Zhang R, Shao J, Li Y *et al.* Inhibition of Dectin-1 Alleviates Neuroinflammatory Injury by Attenuating NLRP3 Inflammasome-Mediated Pyroptosis After Intracerebral Hemorrhage in Mice: Preliminary Study Results. J Inflamm Res*.* 2022; 15: 5917-5933.

8 Chen S, Peng J, Sherchan P, Ma Y, Xiang S, Yan F *et al.* TREM2 activation attenuates neuroinflammation and neuronal apoptosis via PI3K/Akt pathway after intracerebral hemorrhage in mice. J Neuroinflammation*.* 2020; 17: 168.

9 Zhao Y, Xiao Q, Sun T, Yu H & Luo M. Knockdown of LCN2 Attenuates Brain Injury After Intracerebral Hemorrhage via Suppressing Pyroptosis. Neuropsychiatr Dis Treat*.* 2024; 20: 83-99.

10 Guo C, Zhou X, Wang X, Wang H, Liu J, Wang J *et al.* Annao Pingchong decoction alleviate the neurological impairment by attenuating neuroinflammation and apoptosis in intracerebral hemorrhage rats. J Ethnopharmacol*.* 2023; 310: 116298.

11 Ding M, Jin L, Wei B, Cheng W, Liu W, Li X *et al.* Tumor necrosis factor-stimulated gene-6 ameliorates early brain injury after subarachnoid hemorrhage by suppressing NLRC4 inflammasome-mediated astrocyte pyroptosis. Neural Regen Res*.* 2024; 19: 1064-1071.

12 Lei P, Li Z, Hua Q, Song P, Gao L, Zhou L *et al.* Ursolic Acid Alleviates Neuroinflammation after Intracerebral Hemorrhage by Mediating Microglial Pyroptosis via the NF-kappaB/NLRP3/GSDMD Pathway. Int J Mol Sci*.* 2023; 24.

**Supplemental Figure legends**

**Supplementary Figure 1** Schematic diagram of experimental design. (A) Temporal expression trends and cellular localization of CXCR3 and CXCL10. (B) Effects of CXCR3 inhibition on the blood-brain barrier and neurological function. (C) In vivo experiments investigating the impact of CXCL10 activation of CXCR3 on the pyroptosis of cerebral vascular endothelial cells and the BBB disruption. (D) Investigating the cellular source of CXCL10 and its mechanistic role in endothelial cell pyroptosis. (E) Exploration of the effects of cGAS-STING signaling activation on pyroptosis in mouse vascular endothelial cells. (F) In vivo and in vitro experiments examining the effects of inhibiting cGAS and AIM2 signaling on pyroptosis in mouse vascular endothelial cells.

**Supplementary Figure 2** (A) Representative immunofluorescence images of primary astrocytes. (B) Calcein/PI staining representative fluorescence images. (C) Quantitative analysis of average fluorescence intensity of PI positive cells.
