# Supplementary figures and images for: Astrocyte-derived CXCL10 exacerbates endothelial cells pyroptosis and blood–brain barrier disruption via CXCR3/cGAS/AIM2 pathway after intracerebral hemorrhage
Source: Cell Death Discov. 2025 Aug 8;11:373. doi: 10.1038/s41420-025-02658-8 (PMC12334743; doi:10.1038/s41420-025-02658-8)

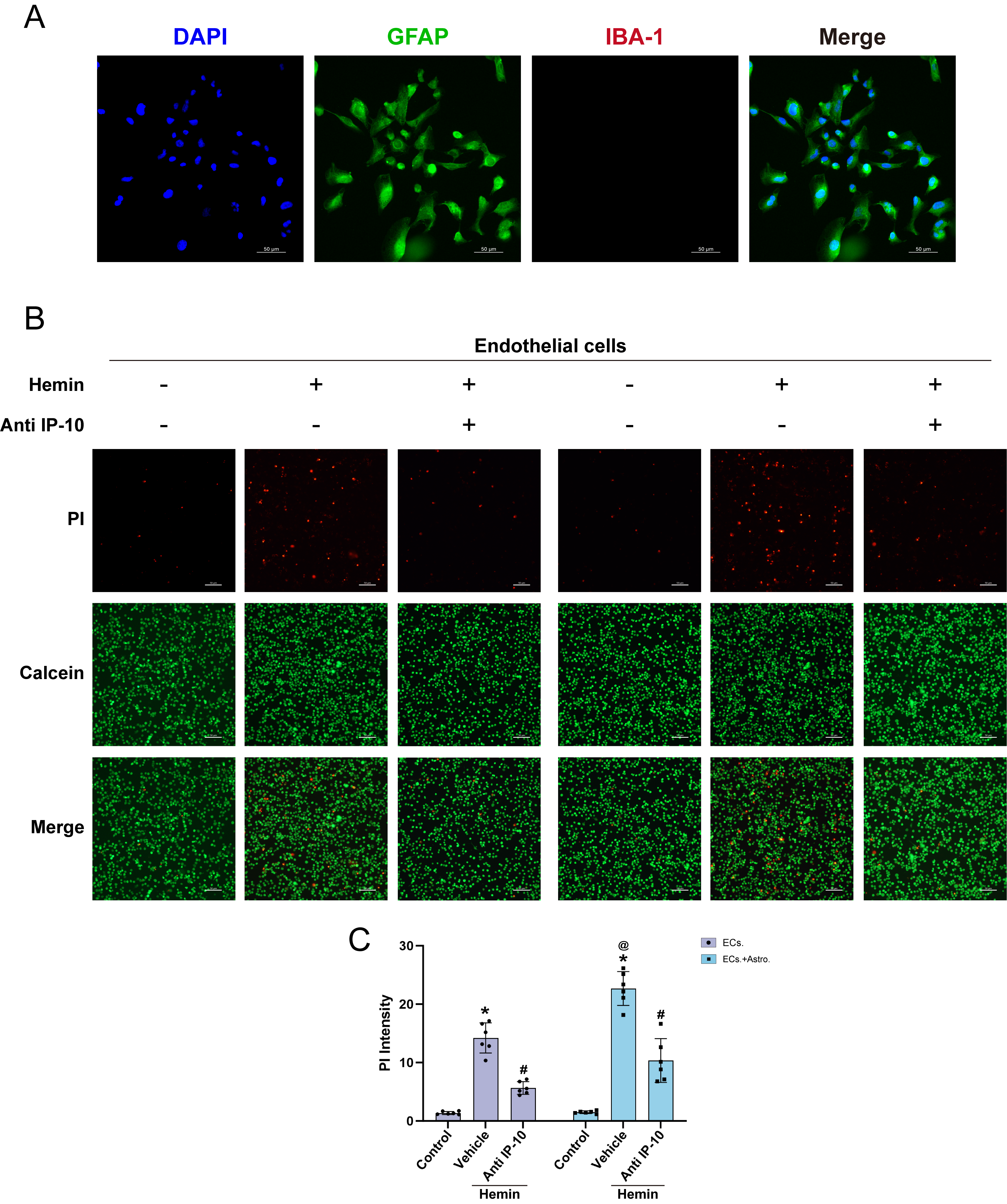

Supplement: Supplementary file 2 — Supplementary Figure 1 [file 41420_2025_2658_MOESM2_ESM.tif]

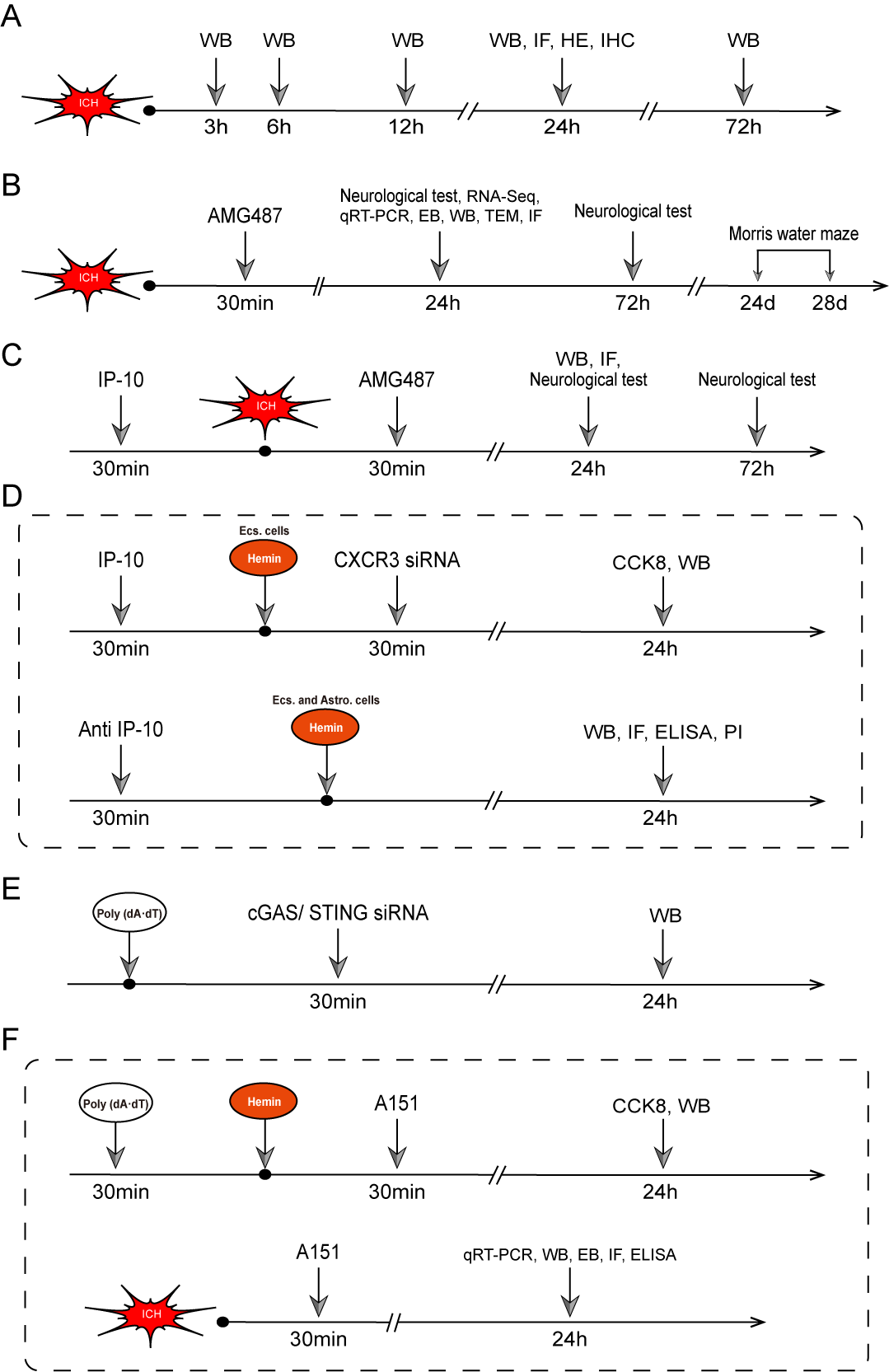

Supplement: Supplementary file 3 — Supplementary Figure 2 [file 41420_2025_2658_MOESM3_ESM.tif]
