# Supplementary material for: Astrocyte-derived CXCL10 exacerbates endothelial cells pyroptosis and blood–brain barrier disruption via CXCR3/cGAS/AIM2 pathway after intracerebral hemorrhage
Source: Cell Death Discov. 2025 Aug 8;11:373. doi: 10.1038/s41420-025-02658-8 (PMC12334743; doi:10.1038/s41420-025-02658-8)
Supplement: Supplementary file 4 — Supplementary Table 1 [file 41420_2025_2658_MOESM4_ESM.docx]

Table 1

Table of antibodies used

| Antibody name | Host spices and clone | Manufacturer | Usage | Dilution ratio | Identifier |
| --- | --- | --- | --- | --- | --- |
| CXCR3 | Rabbit Monoclonal antibody | Proteintech | WB, IF, IHC | WB = 1:2000  IF = 1:100  IHC = 1:50 | 26756-1-AP |
| CXCL9 | Rabbit Monoclonal  antibody | Abcam | WB | 1:1000 | ab320827 |
| CXCL10 | Rabbit Polyclonal antibody | Affinity | WB | 1:1000 | DF6417 |
| CXCL11 | Rabbit Monoclonal  antibody | Abcam | WB | 1:1000 | ab259863 |
| ZO-1 | Rabbit Polyclonal antibody | Proteintech | WB, IF | WB=1:2000  IF=1:100 | 21773-1-AP |
| Occludin | Rabbit Polyclonal antibody | Proteintech | WB | 1:5000 | 27260-1-AP |
| Claudin-5 | Rabbit Polyclonal antibody | Proteintech | WB | 1:5000 | 29767-1-AP |
| cGAS | Rabbit Polyclonal antibody | Proteintech | WB | 1:2000 | 29958-1-AP |
| STING | Rabbit Polyclonal antibody | Proteintech | WB | 1:5000 | 19851-1-AP |
| AIM2 | Rabbit Polyclonal antibody | Proteintech | WB | 1:2000 | 20590-1-AP |
| ASC | Rabbit Polyclonal antibody | Proteintech | WB | 1:5000 | 30641-1-AP |
| Caspase-1 | Rabbit Polyclonal antibody | Affinity | WB | 1:1000 | AF4005 |
| GSDMD | Rabbit Polyclonal antibody | Abcam | WB | WB=1:1000 | ab209845 |
| GSDMD | Rabbit Polyclonal antibody | Proteintech | IF | 1:100 | 20770-1-AP |
| IBA-1 | Mouse Polyclonal antibody | servicebio | IF | 1:100 | GB15105 |
| vWF | Mouse Polyclonal antibody | Proteintech | IF | 1:300 | 66682-1-Ig |
| NeuN | Mouse Polyclonal antibody | Abcam | IF | 1:500 | ab104224 |
| GFAP | Mouse Polyclonal antibody | Affinity | IF | 1:50 | BF0345 |
| CD31 | Rabbit Polyclonal antibody | Proteintech | IF | 1:200 | 80530-1-RR |
| β-actin | Rabbit polyclonal | Beyotime | WB | 1:1000 | AF5003 |
| Rabbit lgG (HPR) | Goat | Beyotime | WB | 1:10000 | A0208 |
| Mouse lgG (HPR) | Goat | Beyotime | WB | 1:10000 | A0216 |
| Goat Anti- Rabbit/Mouse IgG H&L (Alexa Fluor® 488) | Goat | Abcam | IF | 1:1000 | ab150077  ab150113 |
| Goat Anti- Rabbit/Mouse IgG H&L (Alexa Fluor® 647) | Goat | Abcam | IF | 1:1000 | ab150083  ab150115 |
