# Supplementary material for: Astrocyte-derived CXCL10 exacerbates endothelial cells pyroptosis and blood–brain barrier disruption via CXCR3/cGAS/AIM2 pathway after intracerebral hemorrhage
Source: Cell Death Discov. 2025 Aug 8;11:373. doi: 10.1038/s41420-025-02658-8 (PMC12334743; doi:10.1038/s41420-025-02658-8)
Supplement: Supplementary file 5 — Supplementary Table 2 [file 41420_2025_2658_MOESM5_ESM.docx]

Table 2

Primers Used for qRT-PCR

| **Gene** | **Primer sequences, 5′-3′**  **Forward** | **Reverse** |
| --- | --- | --- |
| CXCR3 | TTCCTGCTCCACCTGGCTGTAG | CGCTGATCGTAGTTGGCTGATAGG |
| cGAS | CGGCTGAGTTCCTGAAGATGATGG | CCCTTCCTTCTCCCTCTCCCTTTC |
| STING | AAGAGGAACAGAGGAGGGCGATC | TGGAGCAGAAGAGACAATGAAGAGC |
| AIM2 | ACAGAGCCCTAGAGTAGCCAATCAC | CCCACCTGCCCTCTCTCATAGC |
| ZO-1 | ATTCTCGCTCGGACTCTCCTTCTC | TTTACTATGTGCCCAACAGCAGGTG |
| Claudin-5 | CTGCCTTCCTGGACCACAACATC | GTGAGTGCTACCCGTGCCTTAAC |
| Occludin | ACGGTCCTCCTGGCTCAGTTG | CGGGCATTTCTGGTGGACAAGG |
| GAPDH | AGGTCGGTGTGAACGGATTTG | TGTAGACCATGTAGTTGAGGTGA |
