# Supplementary material for: Astrocyte-derived CXCL10 exacerbates endothelial cells pyroptosis and blood–brain barrier disruption via CXCR3/cGAS/AIM2 pathway after intracerebral hemorrhage
Source: Cell Death Discov. 2025 Aug 8;11:373. doi: 10.1038/s41420-025-02658-8 (PMC12334743; doi:10.1038/s41420-025-02658-8)
Supplement: Supplementary file 6 — Uncropped original Western blot images [file 41420_2025_2658_MOESM6_ESM.pdf]

Figure 1A

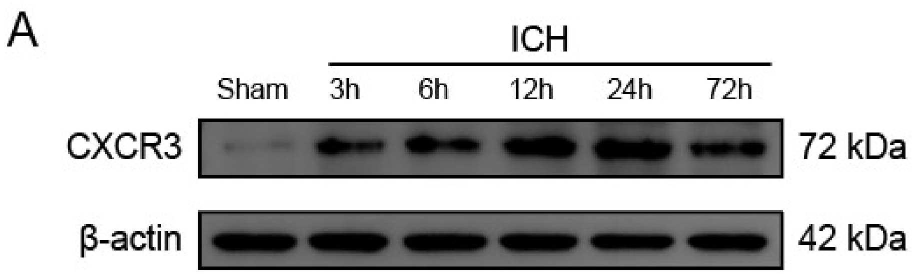
A
ICH
Sham
3h
6h
12h
24h
72h
CXCR3
72 kDa
β-actin
42 kDa

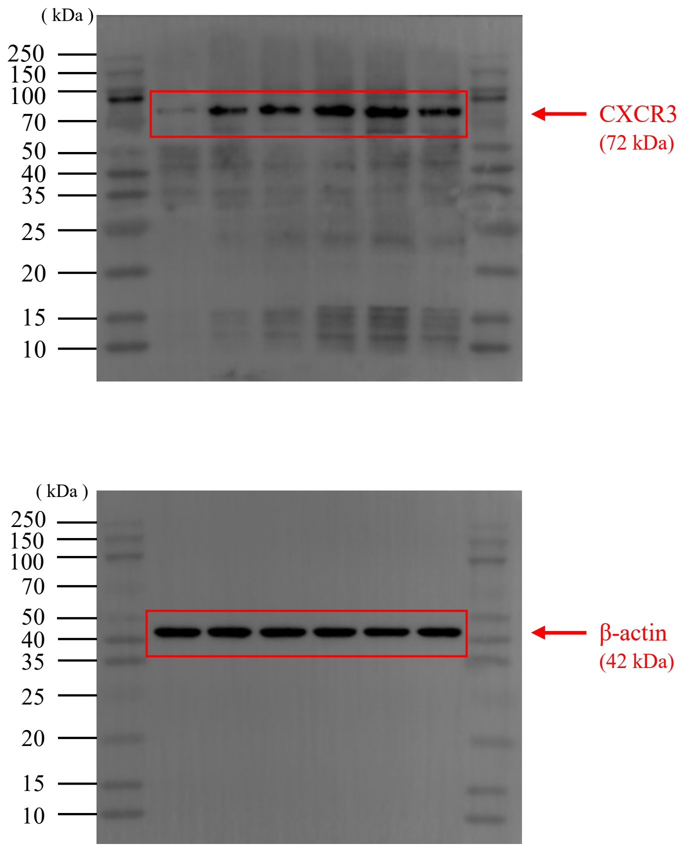
(kDa)
250
150
100
70
50
40
35
25
20
15
10
CXCR3
(72 kDa)
(kDa)
250
150
100
70
50
40
35
25
20
15
10
β-actin
(42 kDa)

Figure 4K

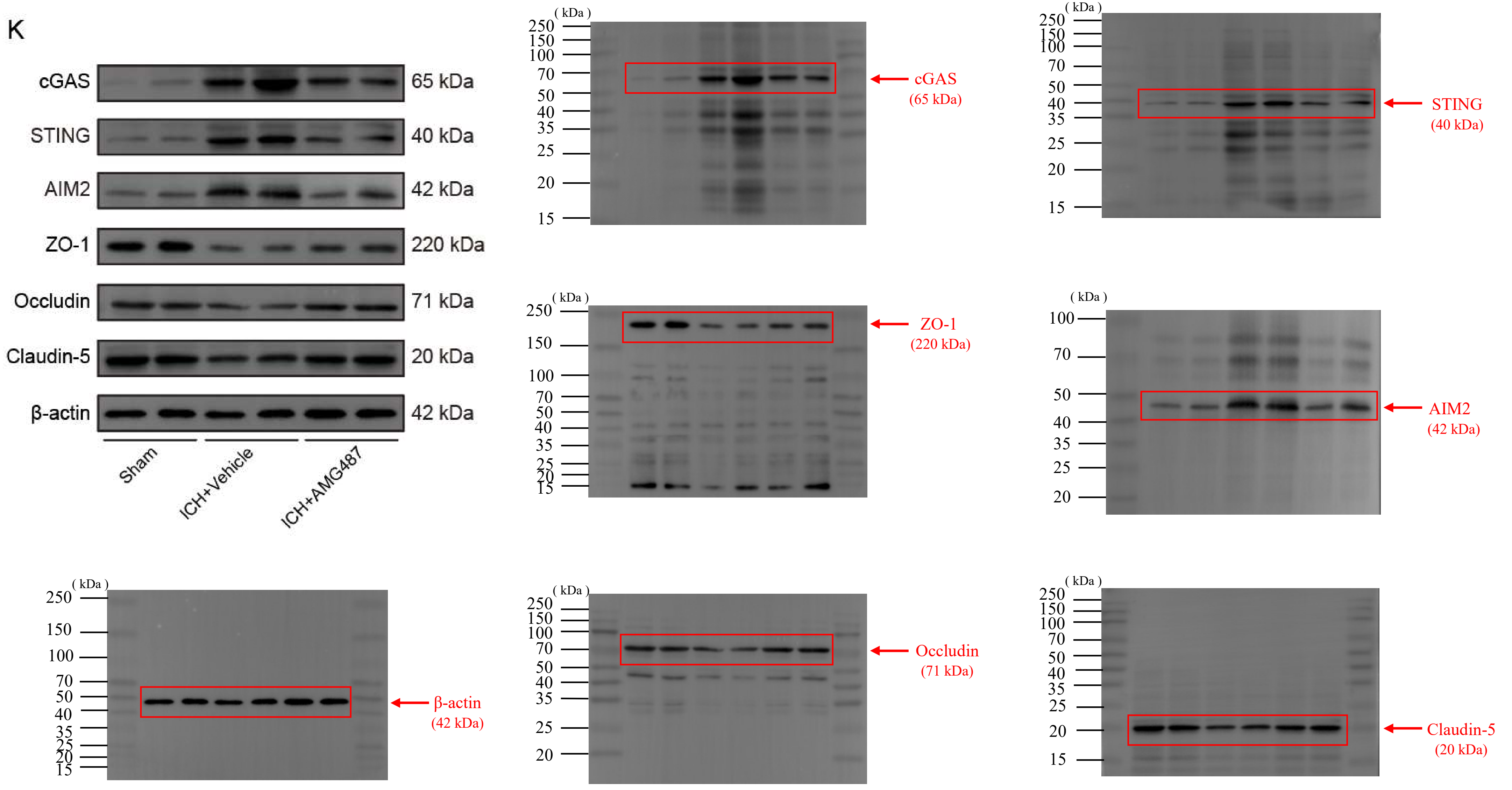
K
cGAS
65 kDa
STING
40 kDa
AIM2
42 kDa
ZO-1
220 kDa
Occludin
71 kDa
Claudin-5
20 kDa
β-actin
42 kDa
Sham
ICH+Vehicle
ICH+AMG487
(kDa)
250
150
100
70
50
40
35
25
20
15
cGAS
(65 kDa)
STING
(40 kDa)
ZO-1
(220 kDa)
AIM2
(42 kDa)
β-actin
(42 kDa)
Occludin
(71 kDa)
Claudin-5
(20 kDa)

Figure 5A

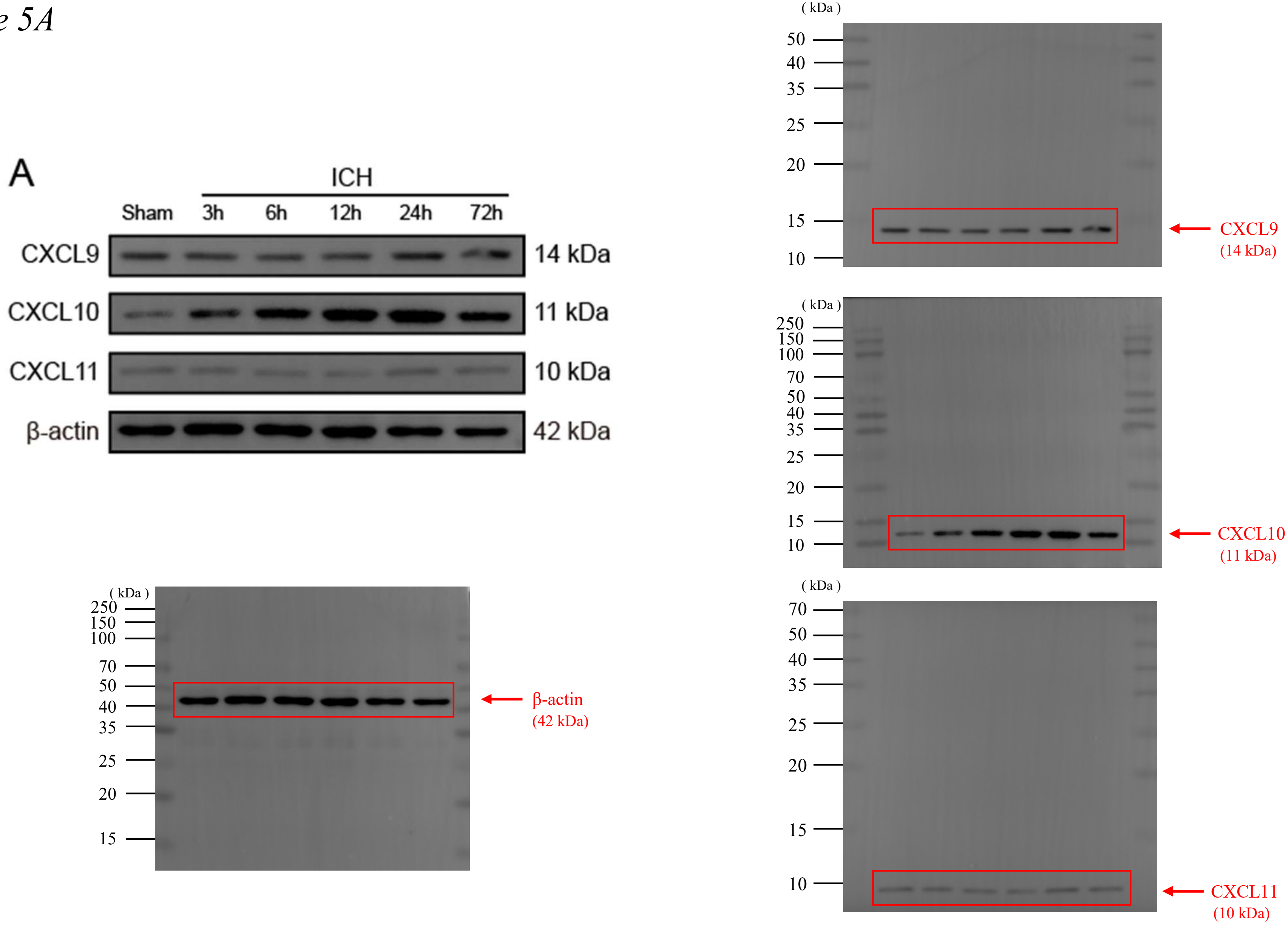
A
ICH
Sham
3h
6h
12h
24h
72h
CXCL9
14 kDa
CXCL10
11 kDa
CXCL11
10 kDa
β-actin
42 kDa
(kDa)
50
40
35
25
20
15
10
CXCL9
(14 kDa)
(kDa)
250
150
100
70
50
40
35
25
20
15
10
CXCL10
(11 kDa)
(kDa)
70
50
40
35
25
20
15
10
CXCL11
(10 kDa)
(kDa)
250
150
100
70
50
40
35
25
20
15
β-actin
(42 kDa)

Figure 6I

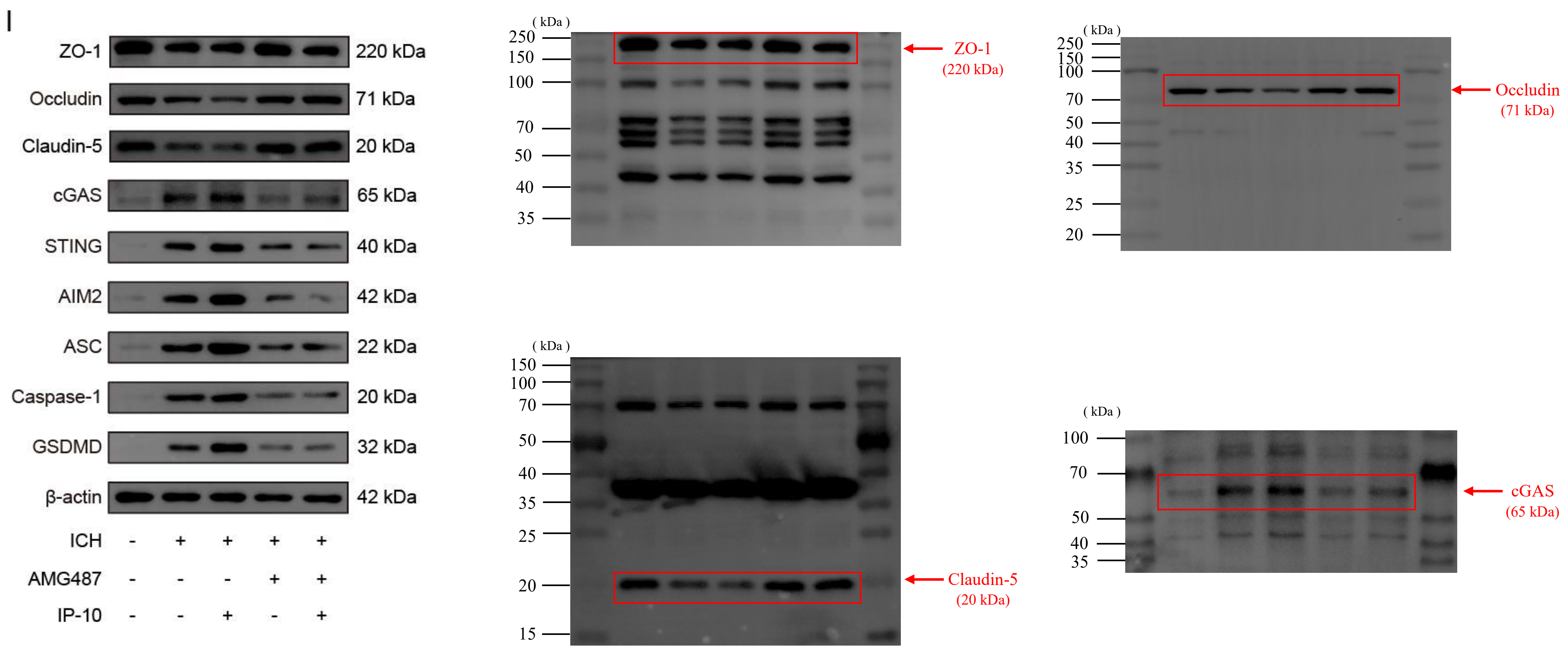
I
ZO-1
220 kDa
Occludin
71 kDa
Claudin-5
20 kDa
cGAS
65 kDa
STING
40 kDa
AIM2
42 kDa
ASC
22 kDa
Caspase-1
20 kDa
GSDMD
32 kDa
β-actin
42 kDa
ICH - + + + +
AMG487 - - - + +
IP-10 - - + - +
(kDa)
250
150
100
70
50
40
35
ZO-1
(220 kDa)
Occludin
(71 kDa)
25
20
Claudin-5
(20 kDa)
15
cGAS
(65 kDa)

Figure 6I

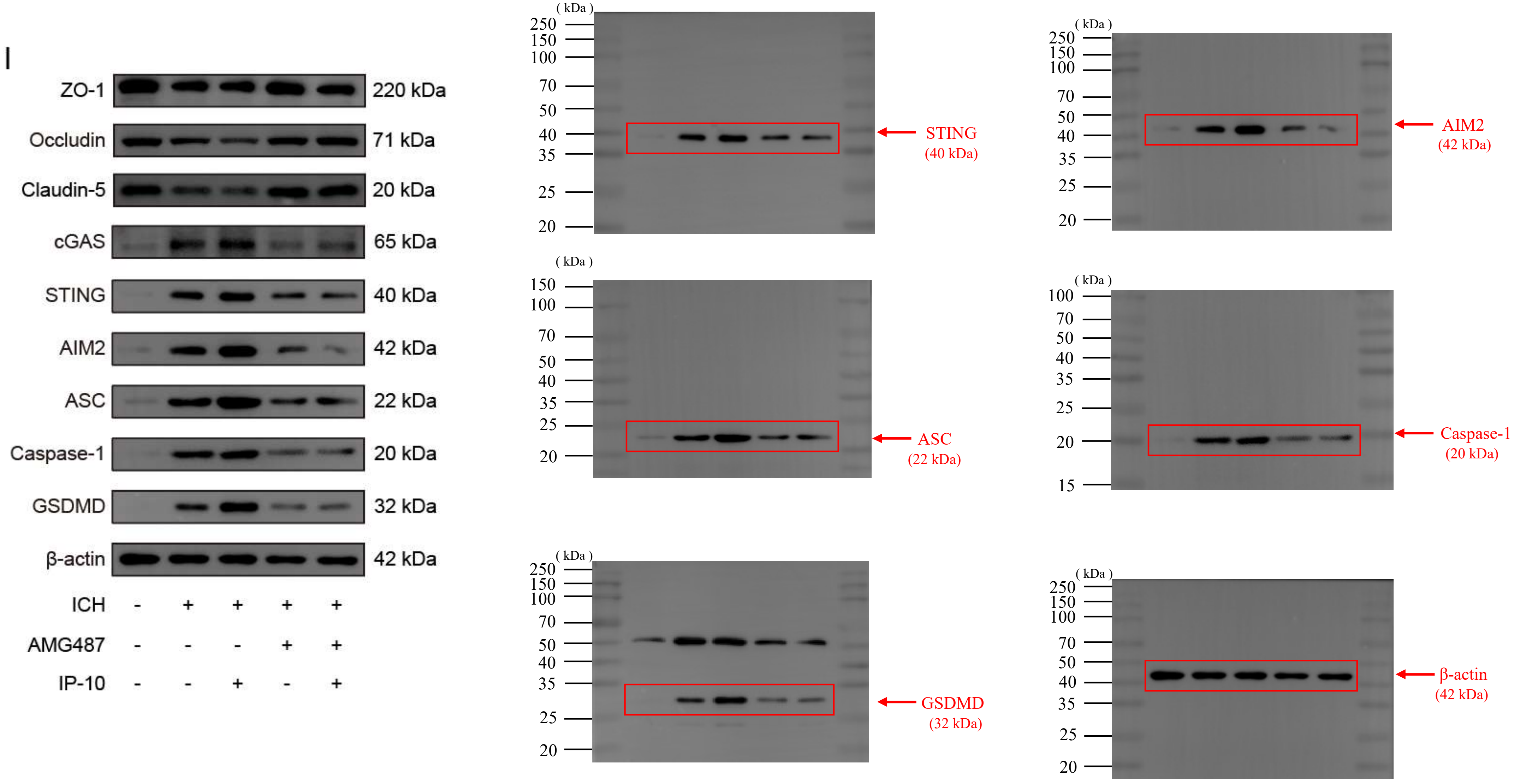
I
ZO-1
220 kDa
Occludin
71 kDa
Claudin-5
20 kDa
cGAS
65 kDa
STING
40 kDa
AIM2
42 kDa
ASC
22 kDa
Caspase-1
20 kDa
GSDMD
32 kDa
β-actin
42 kDa
ICH - + + + +
AMG487 - - - + +
IP-10 - - + - +
(kDa)
STING
(40 kDa)
AIM2
(42 kDa)
ASC
(22 kDa)
Caspase-1
(20 kDa)
GSDMD
(32 kDa)
β-actin
(42 kDa)

Figure 7D

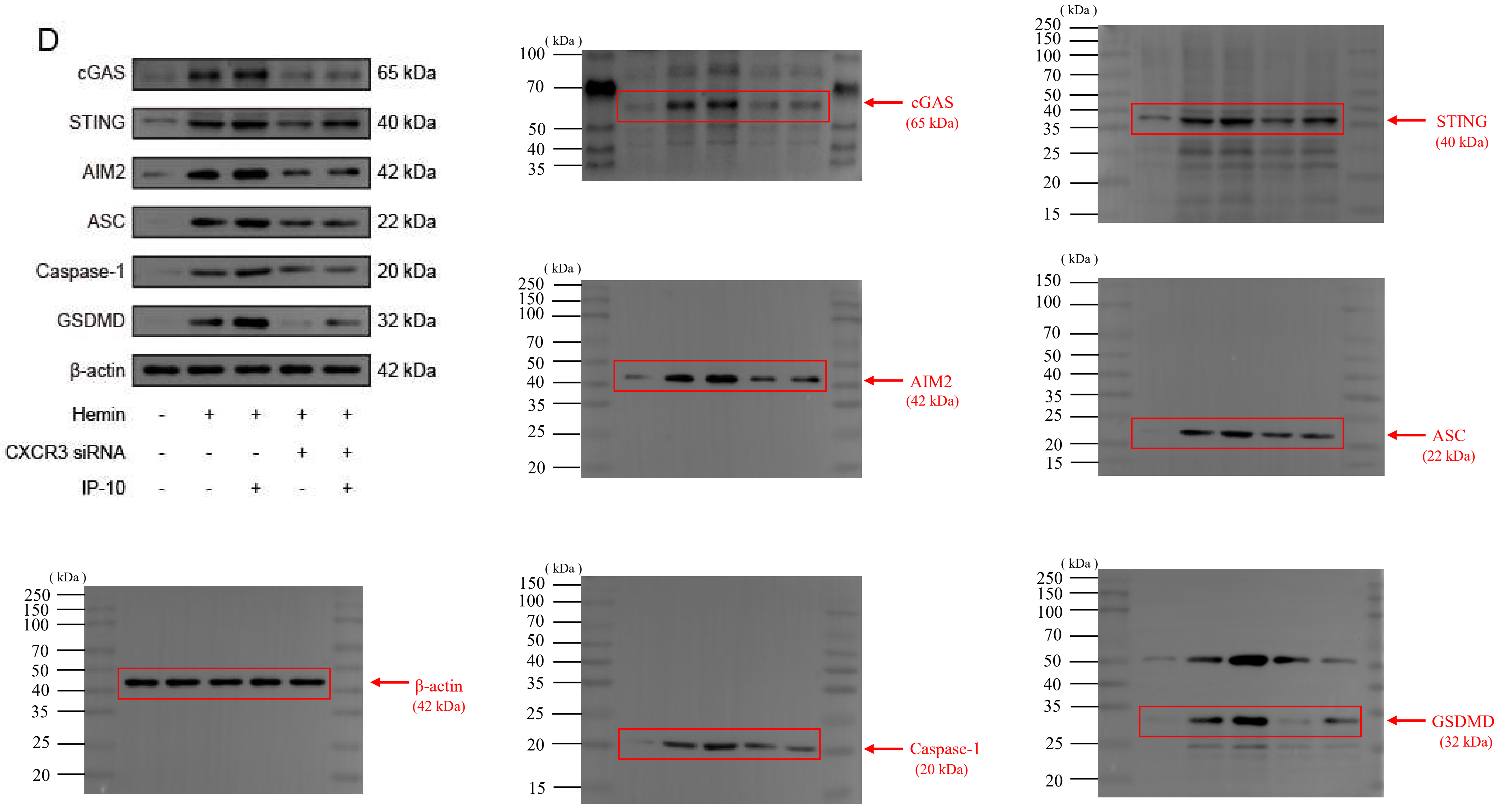
D
cGAS
65 kDa
STING
40 kDa
AIM2
42 kDa
ASC
22 kDa
Caspase-1
20 kDa
GSDMD
32 kDa
β-actin
42 kDa
Hemin - + + + +
CXCR3 siRNA - - - + +
IP-10 - - + - +
(kDa)
100
70
50
40
35
cGAS
(65 kDa)
250
150
100
70
50
40
35
25
20
15
STING
(40 kDa)
AIM2
(42 kDa)
ASC
(22 kDa)
β-actin
(42 kDa)
Caspase-1
(20 kDa)
GSDMD
(32 kDa)

Figure 7M

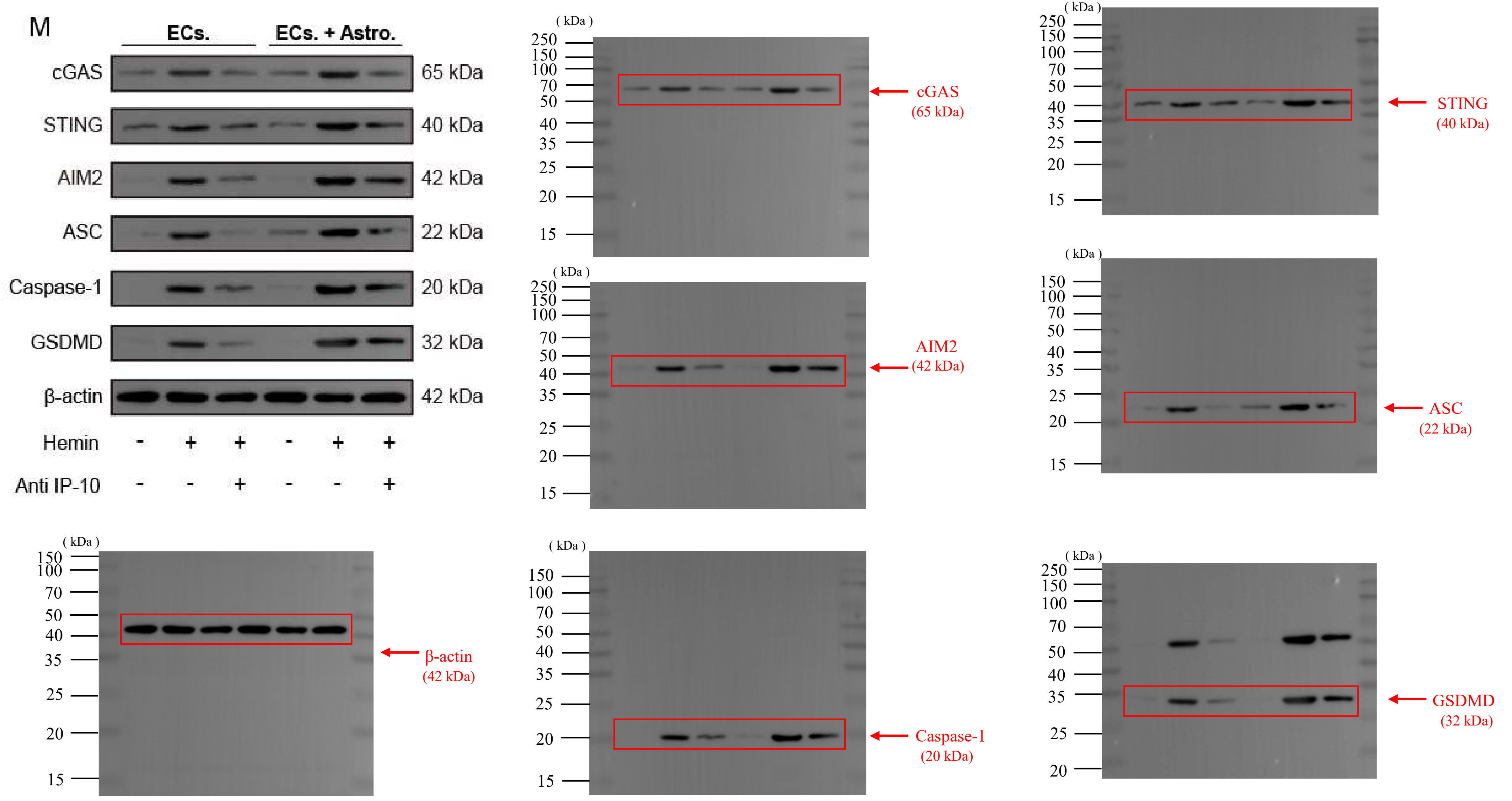
M
ECs.
ECs. + Astro.
cGAS
STING
AIM2
ASC
Caspase-1
GSDMD
β-actin
65 kDa
40 kDa
42 kDa
22 kDa
20 kDa
32 kDa
42 kDa
Hemin - + + - + +
Anti IP-10 - - + - - +
cGAS (65 kDa)
STING (40 kDa)
AIM2 (42 kDa)
ASC (22 kDa)
β-actin (42 kDa)
Caspase-1 (20 kDa)
GSDMD (32 kDa)
(kDa)

Figure 8B

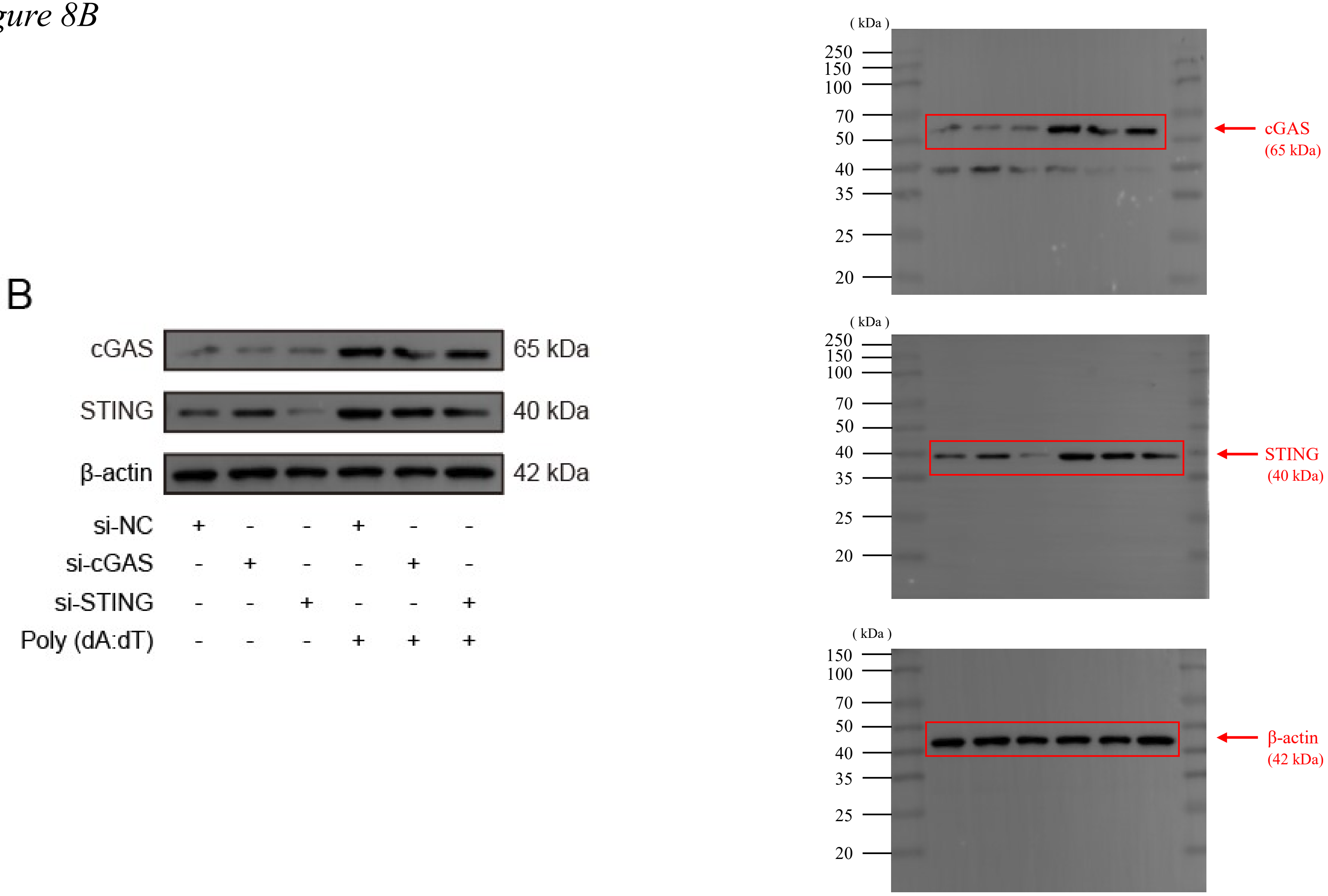
B
cGAS
STING
β-actin
65 kDa
40 kDa
42 kDa
si-NC + - - + - -
si-cGAS - + - - + -
si-STING - - + - - +
Poly (dA:dT) - - - + + +
(kDa)
250
150
100
70
50
40
35
25
20
cGAS
(65 kDa)
STING
(40 kDa)
β-actin
(42 kDa)

Figure 8D

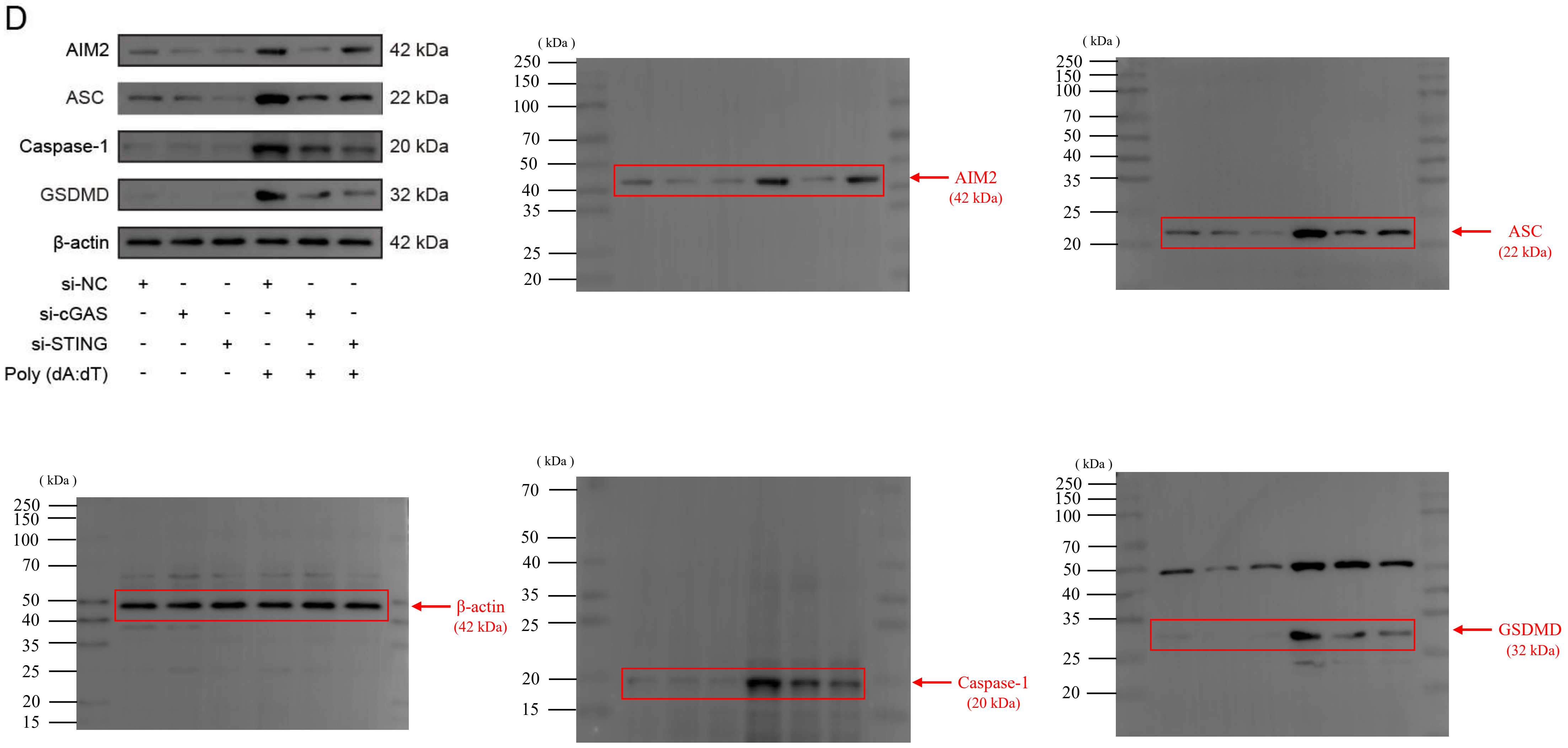
D
AIM2
ASC
Caspase-1
GSDMD
β-actin
42 kDa
22 kDa
20 kDa
32 kDa
42 kDa
si-NC + - - + - -
si-cGAS - + - - + -
si-STING - - + - - +
Poly (dA:dT) - - - + + +
(kDa)
AIM2
(42 kDa)
ASC
(22 kDa)
β-actin
(42 kDa)
Caspase-1
(20 kDa)
GSDMD
(32 kDa)

Figure 8G

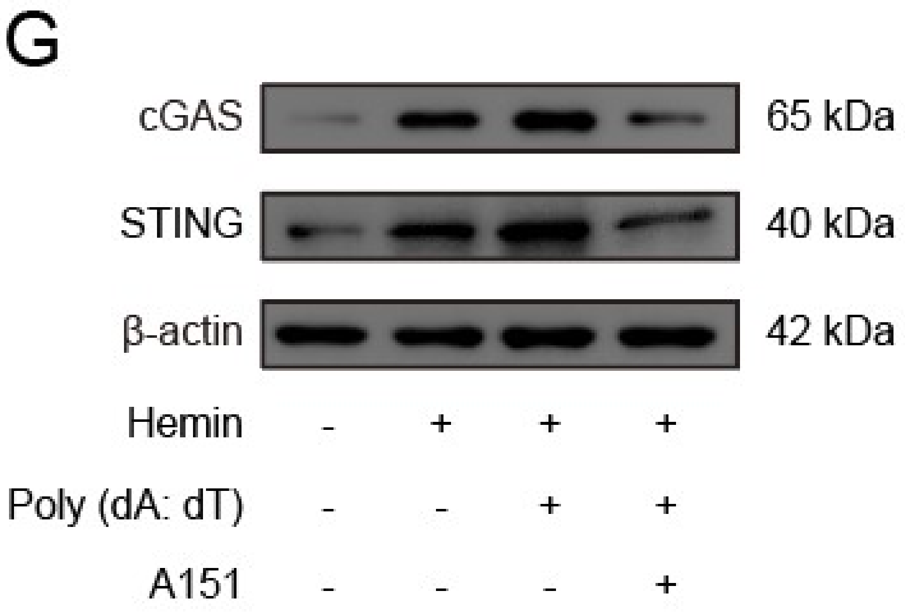
G
cGAS
65 kDa
STING
40 kDa
β-actin
42 kDa
Hemin - + + +
Poly (dA: dT) - - + +
A151 - - - +

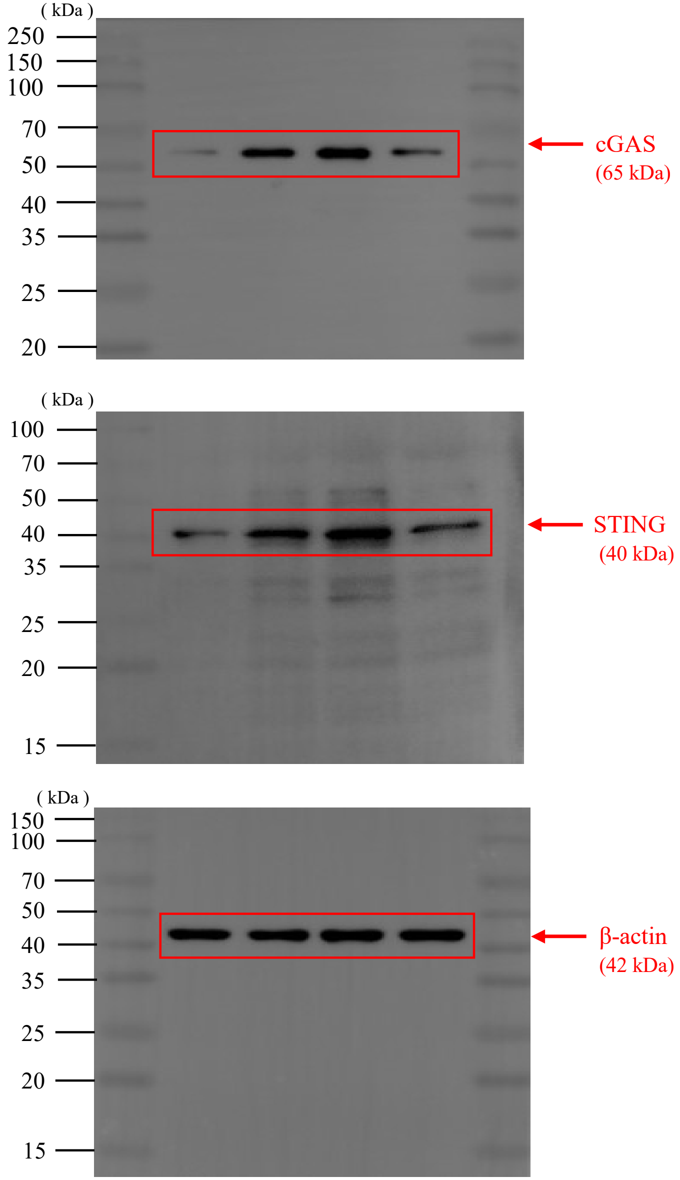
(kDa)
250
150
100
70
50
40
35
25
20
cGAS
(65 kDa)
(kDa)
100
70
50
40
35
25
20
15
STING
(40 kDa)
(kDa)
150
100
70
50
40
35
25
20
15
β-actin
(42 kDa)

Figure 8I

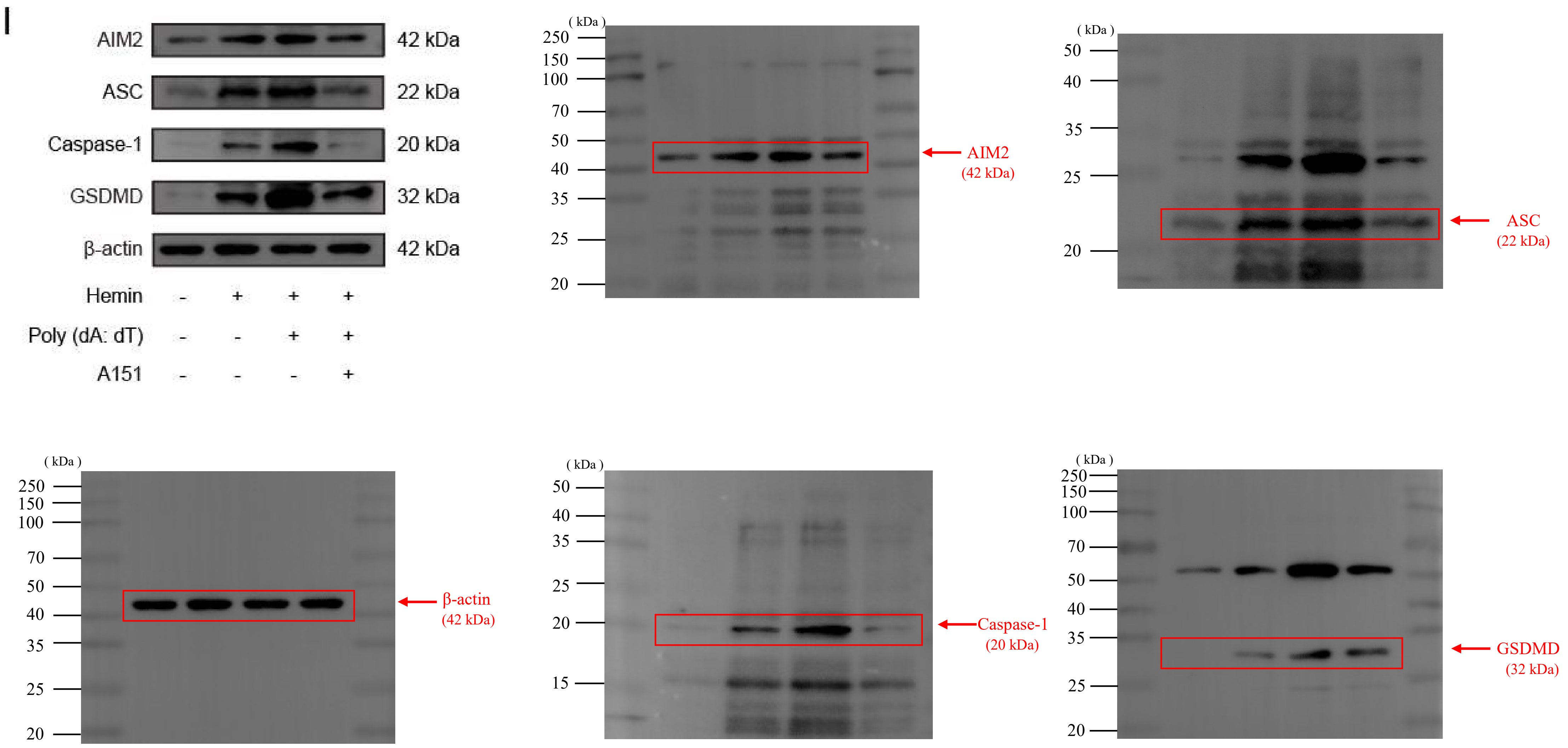

I
AIM2
ASC
Caspase-1
GSDMD
β-actin
42 kDa
22 kDa
20 kDa
32 kDa
42 kDa
Hemin - + + +
Poly (dA: dT) - - + +
A151 - - - +
(kDa)
AIM2
(42 kDa)
ASC
(22 kDa)
β-actin
(42 kDa)
Caspase-1
(20 kDa)
GSDMD
(32 kDa)

Figure 9B

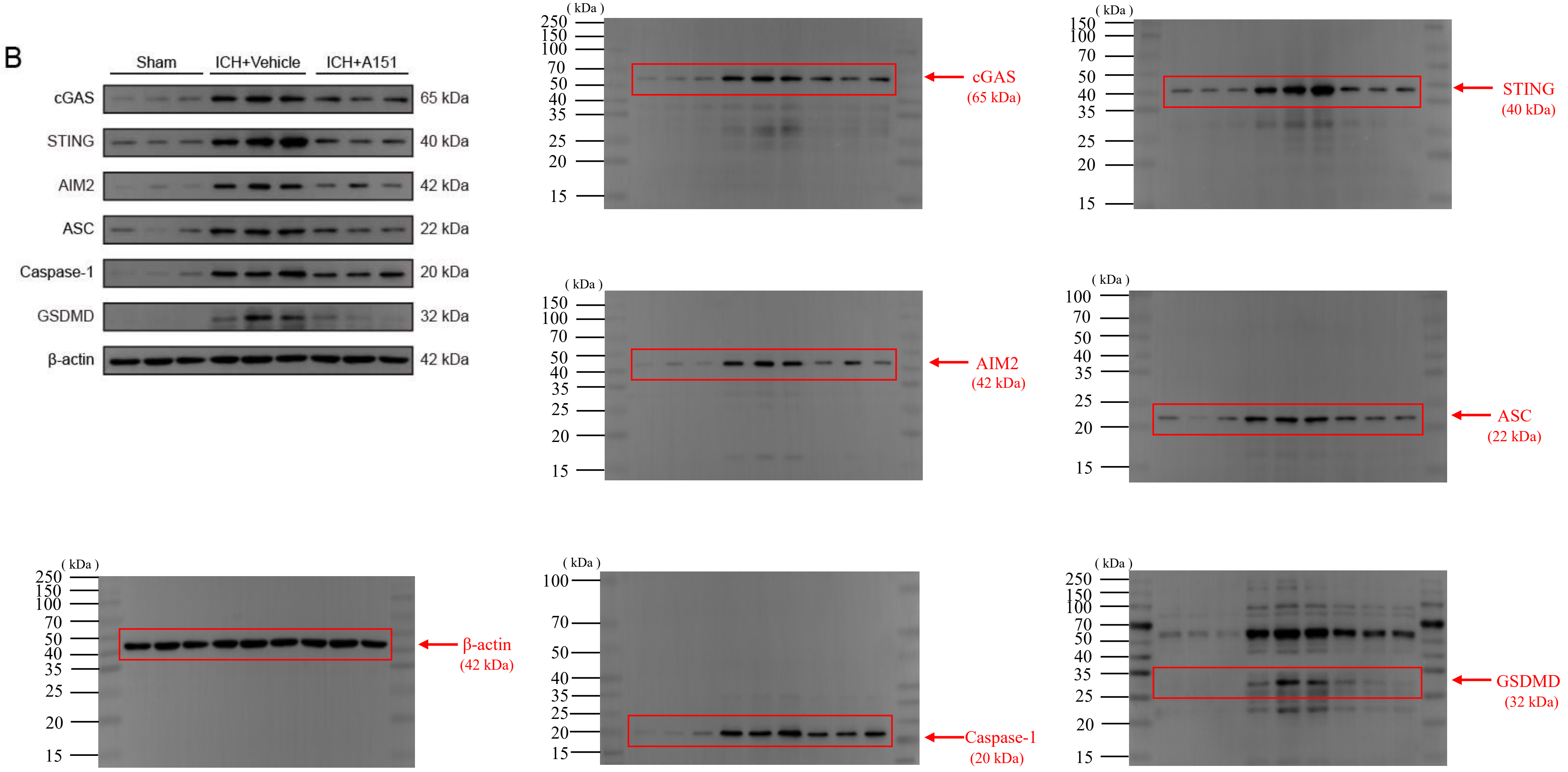
B
Sham
ICH+Vehicle
ICH+A151
cGAS 65 kDa
STING 40 kDa
AIM2 42 kDa
ASC 22 kDa
Caspase-1 20 kDa
GSDMD 32 kDa
β-actin 42 kDa
(kDa)
250
150
100
70
50
40
35
25
20
15
cGAS (65 kDa)
STING (40 kDa)
AIM2 (42 kDa)
ASC (22 kDa)
β-actin (42 kDa)
Caspase-1 (20 kDa)
GSDMD (32 kDa)

Figure 9D

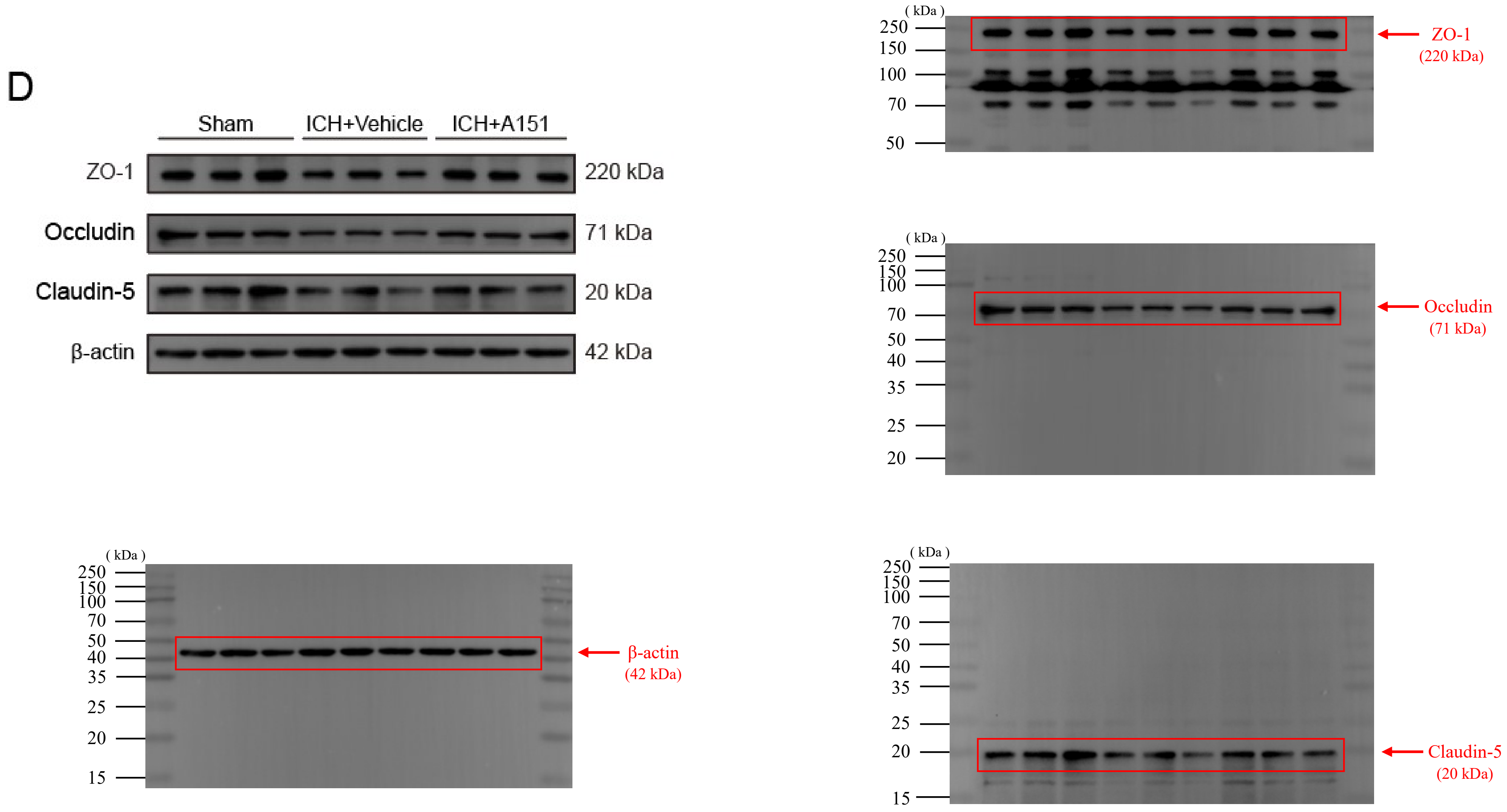
D
Sham
ICH+Vehicle
ICH+A151
ZO-1
Occludin
Claudin-5
β-actin
220 kDa
71 kDa
20 kDa
42 kDa
(kDa)
250
150
100
70
50
ZO-1
(220 kDa)
(kDa)
250
150
100
70
50
40
35
25
20
Occludin
(71 kDa)
(kDa)
250
150
100
70
50
40
35
25
20
15
β-actin
(42 kDa)
(kDa)
250
150
100
70
50
40
35
25
20
15
Claudin-5
(20 kDa)
